# HMGB1 in the mPFC governs comorbid anxiety in neuropathic pain

**DOI:** 10.1186/s10194-022-01475-z

**Published:** 2022-08-16

**Authors:** Yu Du, Ceng-Lin Xu, Jie Yu, Keyue Liu, Shi-Da Lin, Ting-Ting Hu, Feng-Hui Qu, Fang Guo, Guo-Dong Lou, Masahiro Nishibori, Wei-Wei Hu, Zhong Chen, Shi-Hong Zhang

**Affiliations:** 1 Department of Pharmacology and Department of Anesthesiology of the Second Affiliated Hospital, Zhejiang University School of Medicine, Hangzhou, 310058 China; 2 Key Laboratory of Neuropharmacology and Translational Medicine of Zhejiang Province, College of Pharmaceutical Sciences, Zhejiang Chinese Medical University, Hangzhou, 310053 China; 3 Department of Pharmacology, Dentistry and Pharmaceutical Sciences, Okayama University Graduate School of Medicine, Okayama, 700-8558 Japan; 4 Department of Pharmacy, Sir Run Run Shaw Hospital, Zhejiang University School of Medicine, Hangzhou, 310016 China

**Keywords:** HMGB1, Neuroinflammation, mPFC, Neuropathic pain, Comorbid mood disorders, Anxiety

## Abstract

**Background:**

Whether neuroinflammation causes comorbid mood disorders in neuropathic pain remains elusive. Here we investigated the role of high mobility group box 1 protein (HMGB1), a proinflammatory cytokine, in the medial prefrontal cortex (mPFC) in anxiety comorbidity of neuropathic pain.

**Methods:**

Neuropathic pain was induced by partial transection of the infraorbital nerve (p-IONX) or partial sciatic nerve ligation (PSL) in mice and evaluated by measuring nociceptive thresholds to mechanical and heat stimulation. Anxiety-like behaviors were assessed by elevated plus maze, light dark box and open field tests. Aversive or anti-aversive effect was detected by conditioned place preference test. Neuronal activity was evaluated by single-unit and patch clamp recordings. The contribution of mPFC pyramidal neurons to anxiety was further examined by selectively inhibiting them by optogenetics. HMGB1 expression was measured by immunohistochemistry and western blotting. Antagonism of HMGB1 was achieved by injecting anti-HMGB1 monoclonal antibody (mAb) intracerebrally or intraperitoneally.

**Results:**

Anxiety-like behaviors were presented earlier after p-IONX than after PSL. HMGB1 expression was upregulated in the mPFC temporally in parallel to anxiety onset, rather than in other regions associated with anxiety. The upregulation of HMGB1 expression and its translocation from the nucleus to cytoplasm in the mPFC occurred predominantly in neurons and were accompanied with activation of microglia and astrocytes. Infusion of anti-HMGB1 mAb into the mPFC during the early and late phases after either p-IONX or PSL alleviated anxiety-like behaviors and aversion without changing pain sensitization, while local infusion of exogenous ds-HMGB1, the proinflammatory form of HMGB1, into the mPFC induced anxiety and aversion but not pain sensitization in naïve mice. In addition to reversing established pain sensitization and anxiety simultaneously, intraperitoneal injection of anti-HMGB1 mAb reduced HMGB1 upregulation and suppressed the hyperexcitability of layer 2/3 pyramidal neurons in the mPFC after p-IONX. Moreover, optogenetic inhibition of mPFC pyramidal neurons alleviated anxiety in p-IONX mice.

**Conclusion:**

These results demonstrate that HMGB1 in the mPFC drives and maintains anxiety comorbidity in neuropathic pain by increasing the excitability of layer 2/3 pyramidal neurons, and justify antagonism of HMGB1, e.g., neutralization by mAb, as a promising therapeutic strategy for neuropathic pain with anxiety comorbidity.

**Supplementary Information:**

The online version contains supplementary material available at 10.1186/s10194-022-01475-z.

## Background

Clinical studies have shown that anxiety presents in up to 50–60% of patients with neuropathic pain [1, 2]. Because negative affect not only deteriorates pain, but also impedes therapeutic effectiveness [3, 4], interdisciplinary treatment that includes efforts to manage comorbid mood disorders is currently recommended for neuropathic pain [5, 6]. Unfortunately, the first-line analgesics for neuropathic pain, i.e. some antidepressants and antiepileptic drugs, are only effective in some patients at the cost of various adverse effects. Notably, antidepressants cannot evidently reduce anxiety in patients with neuropathic pain [7]. Therefore, the clinical treatment of neuropathic pain with anxiety comorbidity is still challenging and a better understanding of its pathogenesis is urgently required. 

Spinal nerve injury has long been widely adopted to induce neuropathic pain in rodents. Although pain abnormalities manifest in somatic areas rapidly after nerve injury, anxio-depressive behaviors usually present several weeks later with significant discrepancies among studies in terms of onset time and behavioral manifestations [8, 9]. Interestingly, several recent studies reported that rodents exhibit anxiety-like behaviors as early as 2 weeks after infraorbital nerve (ION) injury [10, 11]. The early onset of anxiety comorbidity under orofacial neuropathic pain conditions implies unique pathophysiological changes in the brain. Further study on the mechanisms underlying this phenomenon will provide us with a chance to better understand the comorbid anxiety in neuropathic pain, hence is beneficial for the development of new treatment strategies. 

It has been noticed that inflammation is associated with various types of anxiety disorders [12, 13]. Coincidently, spinal nerve injury is frequently reported to induce neuroinflammatory reactions in multiple brain regions that are implicated in mood disorders including anxiety [14, 15], although the contribution of cerebral neuroinflammation to neuropathic pain and comorbid mood disorders has not been fully understood. By contrast, cerebral neuroinflammation after orofacial nerve injury as well as its association with comorbid mood disorders remain elusive. High mobility group box 1 (HMGB1), a member of damage-associated molecular pattern family, is released either passively from necrotic cells or actively from alive but “stressed” cells after being translocated into the cytoplasm from nucleus [16, 17]. As an alarmin, the extracellular HMGB1 is believed to activate innate and adaptive immunity and drives inflammatory responses via binding to multiple receptors [18, 19]. Previously, we reported that partial transection of ION (p-IONX) in mice evokes orofacial and widespread pain sensitization [20], in which HMGB1 plays crucial roles [21]. However, whether HMGB1 serves as a causative factor for comorbid mood disorders of neuropathic pain has not been studied, although it has been linked to anxiety states under other pathological conditions [22, 23]. 

In the present study, we aimed to investigate the role of cerebral HMGB1 in anxiety comorbidity in neuropathic pain. We found that early-onset anxiety was parallel to the upregulation of HMGB1 in the medial prefrontal cortex (mPFC) in mice after p-IONX. Local administration of monoclonal antibody against HMGB1 (anti-HMGB1 mAb) and exogenous ds-HMGB1 (the pro-inflammatory form of HMGB1) into the mPFC demonstrated the determinant role of HMGB1 in anxiety, which was supported by that optogenetic inhibition of mPFC pyramidal neurons alleviated anxiety. Moreover, the analgesic and anxiolytic effects of intraperitoneal injection of anti-HMGB1 mAb justify antagonism of HMGB1 as a promising therapeutic strategy for neuropathic pain with anxiety comorbidity.

## Methods

### Animals

Male MRL/MPJ and C57BL/6 J mice aged 8–12 weeks at surgery were used in this study. Mice were originally purchased from Jackson Laboratory and multiplied in the animal facility of Zhejiang University. They were housed in ventilated cages in a clean room with controlled humidity (45- 65%) and temperature (22–24℃) on a standard 12-h light–dark cycle (lights on at 8 am). Regular chew and clean water were available ad libitum. All experiments were in accordance with guidelines of The International Association for the Study of Pain [24] and were approved by the Zhejiang University Animal Experimentation Committee. Mice were assigned into different groups randomly. Efforts were made to minimize the animal use and suffering.

### Surgery

#### Partial sciatic nerve ligation (PSL)

Mice were anesthetized by isoflurane inhalation (4% for induction and 2% for maintenance). PSL surgery was done as reported [25]. Briefly, the left sciatic nerve was exposed and under the surgical microscope, an 8/0 silk suture was inserted into the dorsum of the nerve trunk with a 3/8 curved, reversed-cutting needle and was tightly ligated so that the dorsal 1/3–1/2 of the nerve thickness was trapped in the ligature. The sciatic nerve in the sham group was exposed but left intact. The wound was closed in layers.

#### Partial infraorbital nerve transection (p-IONX)

The p-IONX surgery was performed as previously described [20]. In brief, under isoflurane anesthesia, the mouth of the mouse was opened by pulling the lower and upper fore teeth with a rubber thread. Under the surgical microscope, a 2–2.5 mm incision was made from the gingival mucosa of the first molar on the left side to expose the deep branches of the ION. Approximately 1 mm of the nerve fibers was excised with a pair of microsurgical scissors and an absorbent gelatin sponge was placed on the wound. The nerve branches of mice in the sham group were exposed but left uninjured.

### Behavioral assessment

All behavioral tests were conducted during the light phase (from 9 am to 5 pm). Mice were habituated in the testing room for at least 30 min before the test. Examiners were blinded to the groups of mice. The testing room was sound-proof and the illumination intensity was maintained at 80 Lux with temperature at 24–26 °C. After each trial, the mice were put back into their home cages and the experimental apparatus was cleaned with 75% alcohol to eliminate the odor that may affect animal behavior. Anxio-depressive behaviors and evoked pain-like behaviors were assessed on different days after surgery (postoperatively, PO), while the latter were also tested 2 days before surgery (baseline, BL).

### Elevated plus maze (EPM) test

The apparatus used for the EPM test was a cross-shaped device consisting of an intermediate platform region (5 × 0.5 cm^2^) and two pairs of open arms and closed arms (25 × 5 cm^2^) connected thereto. The closed arms are surrounded by high walls (16 cm), whereas the open arms have slight walls (0.5 cm). The entire maze was elevated to a height of 50 cm. The mouse was first placed in the intermediate region at the same position with its head toward open arms, and its behavior in the device was then recorded by a camera for 5 min and the time spent by the animal in each arm was analyzed by ANY-maze [26].

### Light dark box (LDB) test

The apparatus for LDB test was a rectangular box comprising three connected chambers: a light open chamber with white walls (~ 100 Lux, 15 × 20 × 25 cm^3^), a dark covered chamber with black walls (~ 5 Lux, 30 × 20 × 25 cm^3^) and an intermediate small chamber (5 × 20 × 25 cm^3^). The apparatus was placed on the floor. For every trial, the mouse was placed at the same position in the light box. Animal behavior in the apparatus was then recorded for 5 min by a digital camera and the time the animal spent in each box was analyzed by ANY-maze [27].

### Open field test (OFT)

The OFT was performed in an open rectangular box with a bottom edge length of 50 cm and a height of 60 cm. The box was placed directly on the floor. After placing the mouse in the intermediate region of the box, animal behavior was recorded by a camera for 5 min. The bottom of the box was divided into the central (25 × 25 cm^2^) and the rest peripheral region by ANY-maze. The time spent by the animal in each region as well as the moving distance in the box were calculated [28].

### Forced swimming test (FST)

A glass cylinder with a height of 25 cm and a diameter of 10 cm filled with water at 25 °C in a depth of 10 cm was used for FST. The whole test was fulfilled in two days. The mice were placed in the water for 15 min on the first day and 6 min on the next day. The animal behavior was recorded with a camera. After the time had elapsed, the animals were removed from the cylinder and dried before being returned to the home cage. The immobility (or floating) time within the last 4 min on the second day was analyzed by ANY-maze [29].

### Tail suspension test (TST)

The apparatus for the TST was a steel frame with a suspended chain on the middle bar. A black plastic plate was placed behind the chain to provide optical contrast. At the beginning of the test, the mouse was fixed to the chain with a tape at 1.5 cm to the distal end of the tail and was suspended 15 cm above the ground. The camera was then turned on to record the animal behavior for 6 min. Immobility was defined as no physical struggles and the total immobility time from the second to last minute was calculated manually with a stopwatch [30].

### Evoked pain-like behavior test

To test pain-like behaviors evoked by noxious thermal stimulation of the left vibrissal pad, the mouse was placed in a small cage made with metal mesh ceiling and wood bottom (10 × 5 × 5 cm^3^). The laser with a pulse width of 150 ms generated by an infrared diode laser machine (LYPE, China) was shot at the left vibrissal pad with the guidance of a red aiming beam. Evoked pain-like behaviors included scratching the vibrissal pad, shaking head or turning around the body. The laser intensity (A) was started from 15 A and incremented by 1 A. Each intensity was tested 3 times with an interval of at least 5 min. The threshold was defined as the intensity that induced pain-like behaviors at least 2 times out of 3 trails [20]. To test the pain-like behaviors evoked by stimulation of the hind paw, the mouse was placed in a plastic cylinder (height of 9 cm and diameter of 8 cm) with metal mesh bottom. Mechanical stimulation was applied to the plantar surface of the left hind paw by a set of von Frey hairs numbered 1–9 with bending force 0.008, 0.02, 0.03, 0.07, 0.16, 0.4, 0.6, 1.0 and 1.4 g, respectively [31]. The test was started from hair No.5 (0.16 g) and progressed according to an up-down method. Each test constituted a constant number of five stimuli with an interval of at least 5 min. Each stimulus lasted 2 s. A sharp withdrawal or an immediate flinch of the hind paw indicated a positive response. The final number of von Frey hair was determined by adding 0.5 to the number of the fifth test if it evoked responses or reducing 0.5 if it did not. The paw withdrawal threshold (PWT) to mechanical stimulation was calculated by the equation: PWT _force_ = 10^(x*F+B)^ (F is the final number of von Frey hair, x = 0.240, B =  − 2.00). Noxious thermal stimulation was supplied by a laser pulse with a wave width of 200 ms that was shot at the plantar surface of the left hind paw. The laser intensity was increased by 1A step and each intensity was tried 3 times with an interval of at least 5 min. The PWT was defined as the intensity that evoked withdrawal responses at least 2 times out of 3 trails.

### Conditioned place preference (CPP) test

The CPP test was performed in a standard three-box apparatus consisting of two large boxes with the same size (45 × 40 × 35 cm^3^) and a middle connecting channel (40 × 9 × 35 cm^3^). According to previous study [32], the test was divided into consecutive three phases, i.e. habituation or preconditioning, conditioning and testing. During the habituation phase for two days, the mice were placed in the apparatus for 30 min each day and were allowed to move freely with access to all three boxes. On the second day, the movements of each animal in the first 15 min were recorded and analyzed with ANY-maze to verify the absence of preference for each box. Animal spending > 80% or < 20% of the total time in any box were excluded from further testing. A four-day conditioning experiment was then performed. The mice receiving ds-HMGB1 (HMGBiotech Srl, Italy), the pro-inflammatory from of HMGB1 or anti-HMBG1 monoclonal antibody (mAb), the mAb against HMGB1 we developed to neutralize the secreted HMGB1 [33], were placed into the box on one side for 30 min without access to other boxes on the third and the fifth days, while those receiving vehicle were placed into the other side on the fourth and the sixth days for 30 min. On the seventh day, the mice were placed in the middle box with free access to other boxes and their movements were recorded for 15 min. The time that the animal spent in each box and the percentage occupancy (preference) and shifts in occupancy for one side were analyzed by ANY-maze and compared with that on the second day.

### Western blotting

Mice were perfused intracardially with ice-cold 0.9% saline after anesthetized with intraperitoneal (i.p.) injection of pentobarbital (100 mg/kg). The medial prefrontal cortex (mPFC), basolateral amygdala (BLA) and medulla oblongata were removed quickly and frozen in -80 °C fridge. For western blot analysis, frozen tissues were homogenized and lysed in homogenization buffer on ice. Proteins in nucleus and cytoplasm are separated by nuclear and cytoplasmic protein extraction kit (Beyotime, China). Protein concentrations were determined by a bicinchoninic acid assay kit. Protein samples (80 μg) were separated by SDS-PAGE gel electrophoresis and electro-transferred onto a nitrocellulose membrane. After blocking with 5% fat-free milk, the membranes were then incubated with rat anti-HMGB1 mAb (1:1000) and mouse anti-β-tubulin (1:1000; Boster, China) or histone3 (1:1000; CST, USA) polyclonal antibody at 4 °C overnight followed by secondary antibodies conjugated with HRP against either rat or mouse IgG (1:5000; Cell Signaling Technology, USA) for 2 h. Images were captured and quantified by Quantity-One software (Bio-Rad, USA). The ratios between HMGB1 and β-tubulin were calculated and then normalized to the values measured in the control group.

### Immunohistochemistry

Mice were perfused intracardially with ice-cold saline followed by phosphate-buffered 4% paraformaldehyde (pH 7.4) after anesthetized with pentobarbital. The brain was removed and post-fixed overnight in the same fixative and dehydrated in 30% sucrose for 48 h at 4 °C. Coronal brain sections were cut at 20 μm by a cryostat (NX50, Thermo, USA). For immunohistochemical staining, the sections containing the mPFC, BLA, ventral hippocampus (vHPC) and parabrachial nucleus (PBN), four nuclei conventionally implicated in anxiety, were incubated in 0.1% Triton X-100 for 15 min and in 5% donkey serum for 2 h firstly, and then were incubated with rabbit or rat anti-mouse primary antibodies against S100b (1:200, Abcam, UK) /NeuN (1:500, Millipore, USA) /Iba1 (1:500, Wako, Japan) or HMGB1 (1:500) overnight at 4 °C, then with anti-rabbit IgG-Alexa Fluor 488 or anti-rat IgG-Alexa Fluor 594 (1:2000, Invitrogen, USA) for 2 h at room temperature (RT). After repeated washing, the sections were then covered with glass coverslips and fluorescent images were captured by a fluorescence microscope (BX51, Olympus, Japan). The analysis of fluorescence intensity and cell counting were performed by Image J software (NIH, USA). The cytoplasmic translocation of HMGB1 was defined when the intracellular area of HMGB1 immunofluorescence was greater than that of DAPI.

### Agent administration

To implant the canula for intracerebral injection, the mouse was placed on a heating pad and mounted on a stereotaxic apparatus (Stoelting, USA) under anesthesia with sodium pentobarbital (50 mg/kg, i.p.). The skull was exposed and small craniotomies were made over the bilateral mPFC or BLA for guide cannula (0.30 mm in out diameter, RWD Life Science, China) implantation. The coordinates relative to bregma were as follows according to the Paxinos and Franklin (2001) atlas: mPFC (AP: 1.80 mm, ML: ± 0.20 mm, DV: 2.50 mm), BLA (AP: -1.45 mm, ML: ± 2.30 mm, DV: 4.30 mm). The cannulas were held in place by dental cement and the sites of cannula placement were confirmed by histochemistry at the end of all experiments. Ds-HMGB1 (1 μg), anti-HMGB1 mAb (2 μg) or control Ig G (2 μg), all in 1 μl, was infused in 2 min by an injection pump (World Precision Instruments, USA) through a needle that was connected to a 1 μL Hamilton syringe and fit for the cannula. After infusion, the needle was left in place for additional 5 min before slowly withdrawn. For systemic administration injection, anti-HMGB1 mAb (1 mg/kg) or gabapentin (10 or 20 mg/kg) was injected intraperitoneally. Behavioral tests were carried out 1 h after agent administration. All the doses of administered agents were determined by pilot studies.

### Optogenetic experiments

Mice were anesthetized with sodium pentobarbital and mounted in a stereotaxic apparatus. A craniotomy was performed unilaterally and a glass micropipette was introduced into the mPFC (AP: 1.80 mm; ML: 0.20 mm; DV: 2.50 mm) for infusing virus targeting glutamatergic neurons (pAAV-CaMKIIα-eArch3.0-eYFP) in a volume of 100 nl at 0.05 μl/min. The pipette was not removed until 10 min after infusion to allow diffusion of the virus. Animals were kept for 3 weeks to allow the maximal in vivo viral expression before implantation of chronic fiber optic cannula (core diameter 200 μm, 0.22 NA, Newdoon, China) into the mPFC. Behavioral test was carried out another week later. Persistent laser stimulation at 594 nm (5 mW, direct current) was applied through an optogenetic patch cord (Newdoon, China) to inhibit neurons throughout behavioral tests. Mice receiving virus that were connected with the optic fiber but without illumination served as the control for those receiving both virus and illumination. The effective transduction of virus was confirmed by colocalization of eYFP and CaMKIIα, a marker of glutamatergic neurons and by reduction of firing in putative glutamatergic neurons upon laser stimulation.

### In vivo single-unit recordings

Under anesthesia with 20% urethane (1.4 g/kg, i.p.; Sigma-Aldrich, USA), a small craniotomy was performed over the mPFC on D7 PO. The microelectrodes that consisted of 8 channels of wires (25 μm; AM-Systems, USA) with impedances of 1–2 MΩ were lowered into the mPFC by micromanipulator as previously described [34]. Signals were acquired by a multichannel acquisition system (Blackrock Microsystems, USA) at a sampling rate of 30 kHz and high- and low-pass at 250 Hz and 7.5 kHz, respectively, and analyzed by Offline Sorter (Plexon, USA) and NeuroExplorer 4.0 (NEX, USA). Putative glutamatergic neurons were identified according to their wide spike waveform (full width at half maximum ≥ 0.30 ms) and sharp autocorrelation [35, 36].

### Patch clamp electrophysiology

#### Preparation of brain slices

The mouse was anesthetized deeply with isoflurane on day 7 (D7) PO and the brain was quickly removed to icy cold artificial cerebral spinal fluid (ACSF) oxygenated with 95% O_2_ and 5% CO_2_. The brain slices in 300 mm containing the mPFC were obtained by a vibratome (VT1000, Leica Instruments, Germany). The slices were then incubated for at least 30 min at 33 °C and another 1 h at RT in oxygenated ACSF. Then the slices were transferred to a recording chamber and were continuously perfused with oxygenated ASCF at a rate of 3–4 ml/min before electrophysiological recordings at RT. For action potential recordings, the ACSF contained (in mM): 120 NaCl, 11 Dextrose, 2.5 KCl, 1.28 MgSO_4_, 3.3 CaCl_2_, 1 NaH_2_PO_4_, and 14.3 NaHCO_3_, with pH at 7.4 and osmolarity at 310.5 mOsm. For spontaneous post-synaptic current recordings, a low divalent ion ACSF containing (in mM): 125 NaCl, 3.5 KCl, 1.25 NaH_2_PO_4_, 0.5 MgCl_2_, 26 NaHCO_3_, 25 Dextrose, and 1 CaCl_2_, with pH at 7.4 and osmolarity at 310.5 mOsm was used.

#### Whole-cell patch-clamp recordings

Pyramidal neurons of the mPFC slices (coordinates: AP 1.70 mm, ML ± 0.20 mm or ± 0.20 mm, DV 2.10 mm) were visualized and recorded under an infrared differential interference contrast video microscopy mounted on an upright microscope (FN1, Nikon, Japan) equipped with a 340/0.80 water-immersion objective and a charge-coupled device camera (Clara-E, Andor Technology, UK). To record action potentials, pipettes with resistance of 5–10 MΩ and outer diameter of 1.5 mm were filled with a K^+^-based recording solution containing (in mM): 140 K-gluconate, 5 NaCl, 0.2 EGTA, 2 Mg-ATP and 10 HEPES. Stepped currents (0–100 pA, 5 pA per step) were injected into neurons to elicit action potentials. To record spontaneous excitatory and inhibitory post-synaptic currents (sEPSCs and sIPSCs), cesium-based recording solution containing (in mM): 100 CsCH3SO3, 20 KCl, 10 HEPES, 4 Mg-ATP, 0.3 Tris-GTP, 7 Tris2-Phosphocreatine, and 3 QX-314) was used. The holding potential was -60 mV and + 10 mV for recording sEPSCs and sIPSCs, respectively. The signals were amplified by the amplifier (EPC10, HEKA Instruments, Germany), and digitized at 10 kHz. The lowpass filter was set at 2.8 kHz. If the series resistance changed more than 20% during the recordings, the neuron was immediately abandoned. Data were further and analyzed with MiniAnalysis Program (Synatosoft Inc, USA) and Clampfit 10.7 software (Molecular Devices, USA) to provide spreadsheets for the generation of cumulative probability plots. The amplitude and interevent interval of post-synaptic currents were collected. The ratio of charge transfer of sEPSCs (∣CsEPSC∣) and sIPSCs (CsIPSC) was defined as EI ratio.

### Statistical analysis

All data are expressed as the mean ± SEM. The required sample sizes were estimated based on our experience and Power analysis was used to justify the sample size. Statistical analysis was conducted by GraphPad Prism 8.0 (GraphPad Software, USA). Shapiro–Wilk test was used to assess whether the data followed a normal distribution. If the data was normally distributed, two-tailed paired or unpaired Student’s *t* test was used for comparison between two groups; if not, Mann–Whitney test was used instead. One-way analysis of variance (ANOVA) with Dunnett or Tukey *post hoc* test or Kruskal–Wallis test with Dunn’s *post hoc* was used for comparison of more than two groups with one factor. When comparing thresholds to thermal or mechanical stimulation among groups, two-way ANOVA with Bonferroni *post hoc* multiple comparisons test was used. The significance level was set at *P* < 0.05.

## Results

### Early onset anxiety was induced by p-IONX

**Fig. 1:**
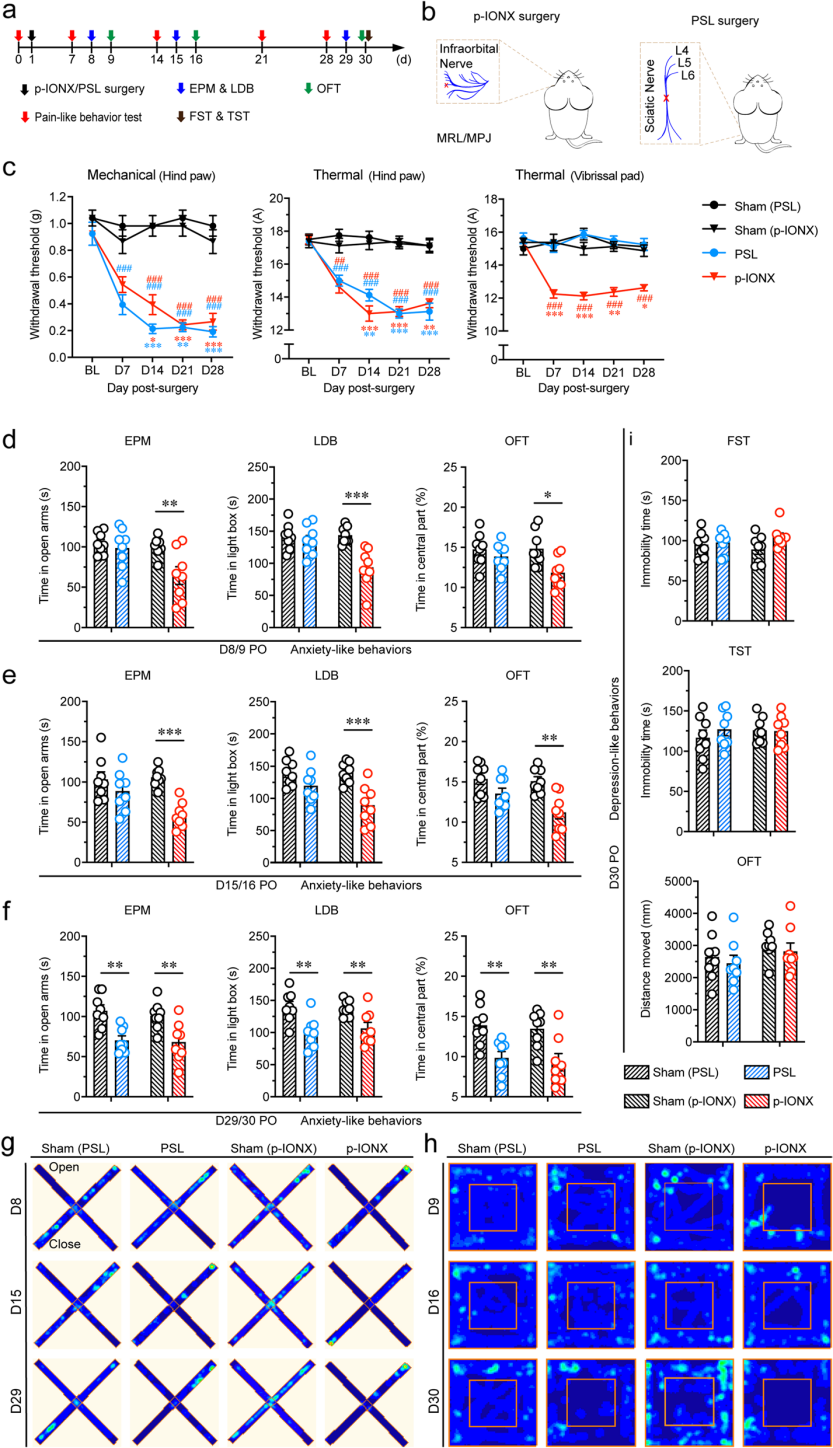
Early onset anxiety was induced by p-IONX. a Schedule of experiment procedures. **b** Schematic of p-IONX and PSL surgery on the left side. **c** Paw withdrawal thresholds to mechanical stimulation and noxious heat stimulation in the ipsilateral hind paw and head withdrawal threshold to noxious heat stimulation in the ipsilateral vibrissal pad before and after surgery. *, ** and *** indicate *P* < 0.05, 0.01 and 0.001, respectively, compared with the baseline (BL), Kruskal–Wallis test with repeated measures and Dunn’s *post hoc*. ## and ### indicate *P* < 0.01 and 0.001, respectively, compared with the respective sham group at the same time points, two-way ANOVA with Bonferroni *post hoc*. **d-f** Anxiety-like behaviors measured by EPM, LDB and OFT tests on D8/9 (**d**), D15/16 (**e**) and D29/30 (**f**) after surgery. *, ** and *** indicate *P* < 0.05, 0.01 and 0.001, respectively, compared with the sham group, unpaired *t* test. **g, h** Representative heat maps from the sham, PSL and p-IONX groups in EPM (**g**) and OFT (**h**) tests. **i** Depression-like behaviors measured by TST, FST and OFT on D30 after surgery. *n* = 8/group

As reported [20, 21, 25, 37], MRL/MPJ mice showed decreased thresholds to mechanical and heat stimulation at the hind paw in both p-IONX and PSL groups and decreased thermal threshold at the vibrissal pad in the p-IONX group, which lasted at least 4 weeks PO (Fig. 1a-c). These results verified that PSL induces somatic, while p-IONX induces widespread (somatic in addition to orofacial) neuropathic pain. Moreover, mice in the p-IONX group displayed anxiety-like behaviors as early as one week after surgery, evidenced by less spent time in the open arm, the dark box and the center zone in the EPM, LDB and OFT tests, respectively. These manifestations maintained up to 4 weeks PO, when mice in the PSL group started to show such anxiety-like behaviors (Fig. 1d-h). By contrast, mice either in the p-IONX or PSL group did not show depression-like behaviors until 4 weeks after surgery, as indicated by unchanged immobility time in the TST and FST as well as unchanged distance moved in the OFT (Fig. 1i). In addition, we found that anxiety-like behaviors were observed in the LDB test in C57BL/6 J mice 2 weeks after p-IONX (Supplementary Fig. 1a-c), which was also earlier than reported after somatic nerve injury [8–10, 38]. These results together demonstrate that early onset anxiety develops under the condition of widespread pain sensitization after trigeminal nerve injury.

### HMGB1 expression in the mPFC was increased in parallel to comorbid anxiety in neuropathic pain

**Fig. 2:**
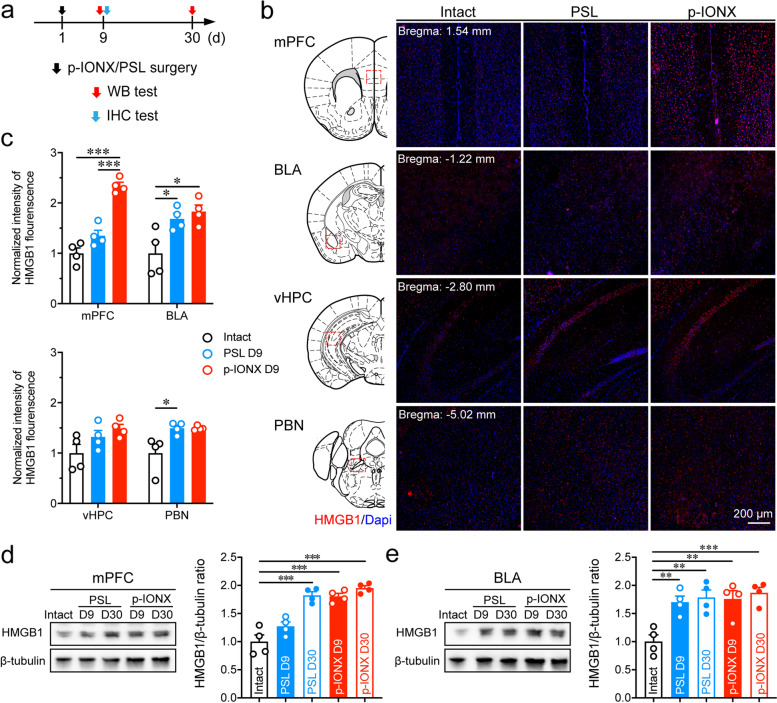
HMGB1 expression in the mPFC was increased temporally in parallel to comorbid anxiety in neuropathic pain. **a** Schedule of experimental procedures. **b** Representative photomicrographs of HMGB1 immunostaining in the mPFC, BLA, vHPC and PBN. Scale bar, 200 μm. **c** Quantification of HMGB1 fluorescence intensity in the mPFC, BLA, vHPC and PBN on D9 PO. **d, e** Representative images of protein bands in western blotting (left) and quantification of HMGB1 expression (right) in the mPFC (**d**) and BLA (**e**) on D9 and D30 PO. *, ** and *** indicate *P* < 0.05, 0.01 and 0.001, respectively, compared with the indicated groups, ordinary one-way ANOVA with Tukey *post hoc* or Kruskal–Wallis test with Dunn’s *post hoc* in (**c**) and ordinary one-way ANOVA with Dunnett *post hoc* in (**d**) and (**e**). *n* = 4/group

To investigate whether HMGB1 is involved in comorbid anxiety in neuropathic pain, the expression of HMGB1 in the brain of MRL/MPJ mice was measured by immunohistochemistry on D9 PO when anxiety-like behaviors were exhibited in the p-IONX but not PSL group (Fig. 2a). We found that HMGB1 expression was increased in many brain regions, such as the cortex, thalamus, hypothalamus and amygdala after p-IONX (Supplementary Fig. 2). The expression in four anxiety-related nuclei, the mPFC, BLA, vHPC and PBN was evaluated semi-quantitatively by measuring the fluorescence intensity and compared between the p-IONX and PSL group. We found that HMGB1 expression was remarkably elevated in the mPFC in the p-IONX group but not in the PSL group, whereas that in the BLA and PBN was up-regulated in both groups without differences, and was unchanged in the vHPC (Fig. 2b and c). The expression changes in the mPFC and BLA were further verified by western blotting (Fig. 2d and e). Interestingly, when anxiety-like behaviors were exhibited on D30 PO in PSL mice, a significant upregulation of HMGB1 expression in the mPFC was also detected (Fig. 2d). By contrast, the expression in the BLA was increased without differences between p-IONX and PSL groups (Fig. 2e). HMGB1 upregulation in the mPFC was also observed in C57BL/6 J mice 2 weeks after p-IONX when anxiety was detected (Supplementary Fig. 1d and e). These results demonstrate that HMGB1 upregulation in the mPFC temporally parallels the onset of comorbid anxiety in neuropathic pain.

### HMGB1 was released mainly by neurons in the mPFC after p-IONX

**Fig. 3:**
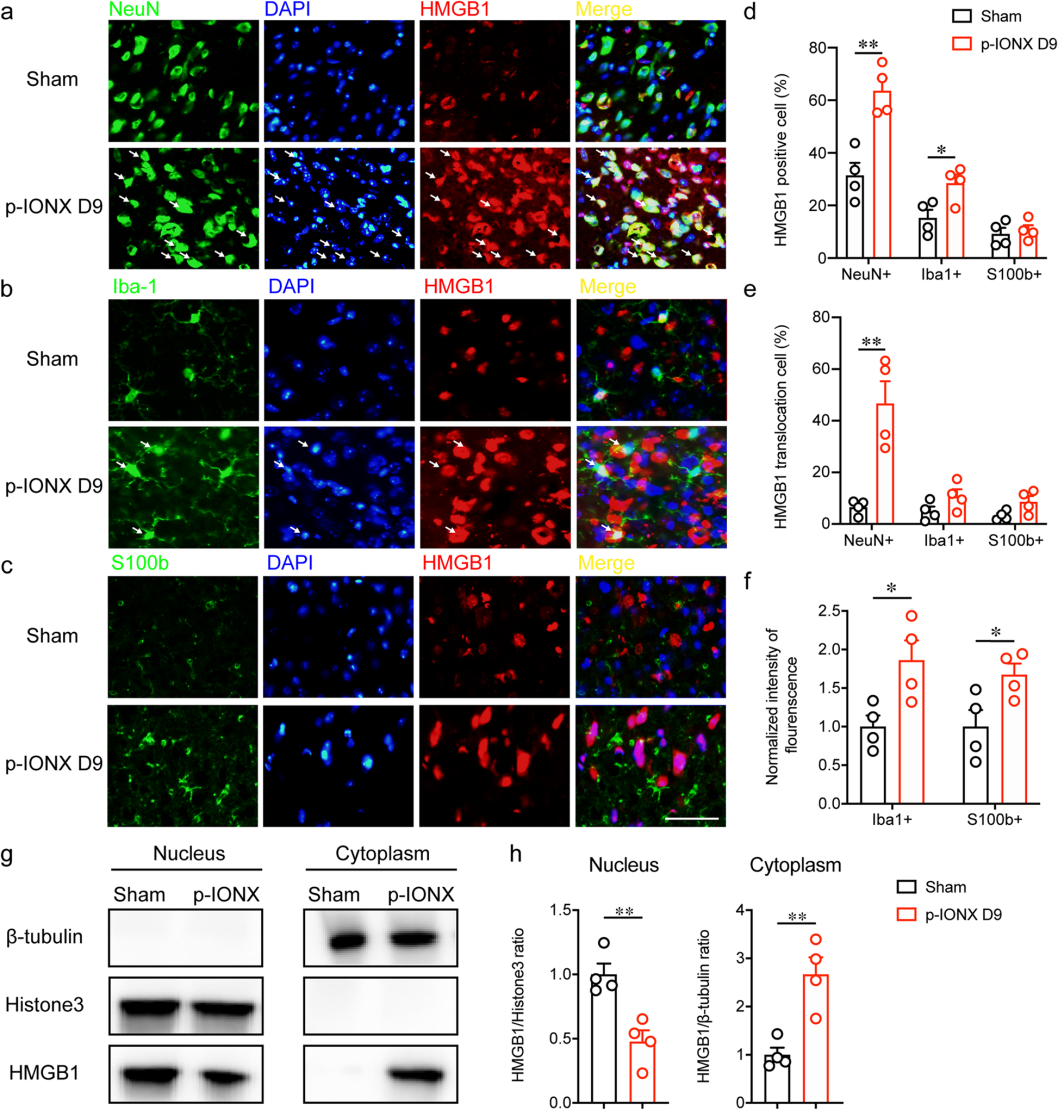
Cellular and subcellular distribution of HMGB1 in the mPFC after p-IONX in MRL/MPJ mice**. a-c** Representative photomicrographs indicating the cellular distribution of HMGB1 in neurons (NeuN^+^, **a**), microglia (Iba1^+^, **b**) and astrocytes (S100b^+^, **c**) in the mPFC on D9 PO. DAPI was used to label nuclei. Scale bar, 50 μm. **d** Percentage of HMGB1-positive cells in neurons (NeuN^+^), microglia (Iba1^+^) and astrocytes (S100b^+^) on D9 PO in the mPFC. **e** Percentage of cells with HMGB1 cytoplasmic translocation in neurons (NeuN^+^), astrocytes (S100b^+^) and microglia (Iba1^+^) on D9 PO in the mPFC. **f** Quantification of Iba1 and S100b fluorescence intensity in the mPFC. **g-h** Representative images of western blot bands (**g**) and quantification of HMGB1 (**h**) in nuclear and cytoplasmic fractions from the mPFC on D9 PO. * and ** indicate *P* < 0.05 and 0.01, respectively, compared with the indicated groups, unpaired *t* test. *n* = 4/group

To identify the cellular sources of HMGB1 upregulation and release, HMGB1 was separately coimmunostained with the markers of neurons (NeuN^+^), microglia (Iba1^+^) and astrocytes (S100b^+^) in the mPFC. The percentages of HMGB1-positive neurons and microglia, but not astrocytes were markedly increased on D9 after p-IONX (Fig. 3a-d). HMGB1 was mainly located in the nuclei in the sham group of mice, but translocated into the cytoplasm after p-IONX, which predominantly occurred in neurons (Fig. 3a-c and e). In addition, microglia and astrocytes in the mPFC presented morphological changes indicative of activation and enhanced fluorescence intensity of Iba1 and S100b (Fig. 3b, c and 

f). The translocation of HMGB1 was further verified by western blotting to quantify the amounts in nuclear and cytoplasmic compartments (Fig. 3g and h). These results demonstrate that HMGB1 upregulation and release in the mPFC after p-IONX mainly occurs in neurons and is accompanied with glial activation.

### HMGB1 upregulation in the mPFC drove anxiety and aversion but not pain sensitization after p-IONX

**Fig. 4:**
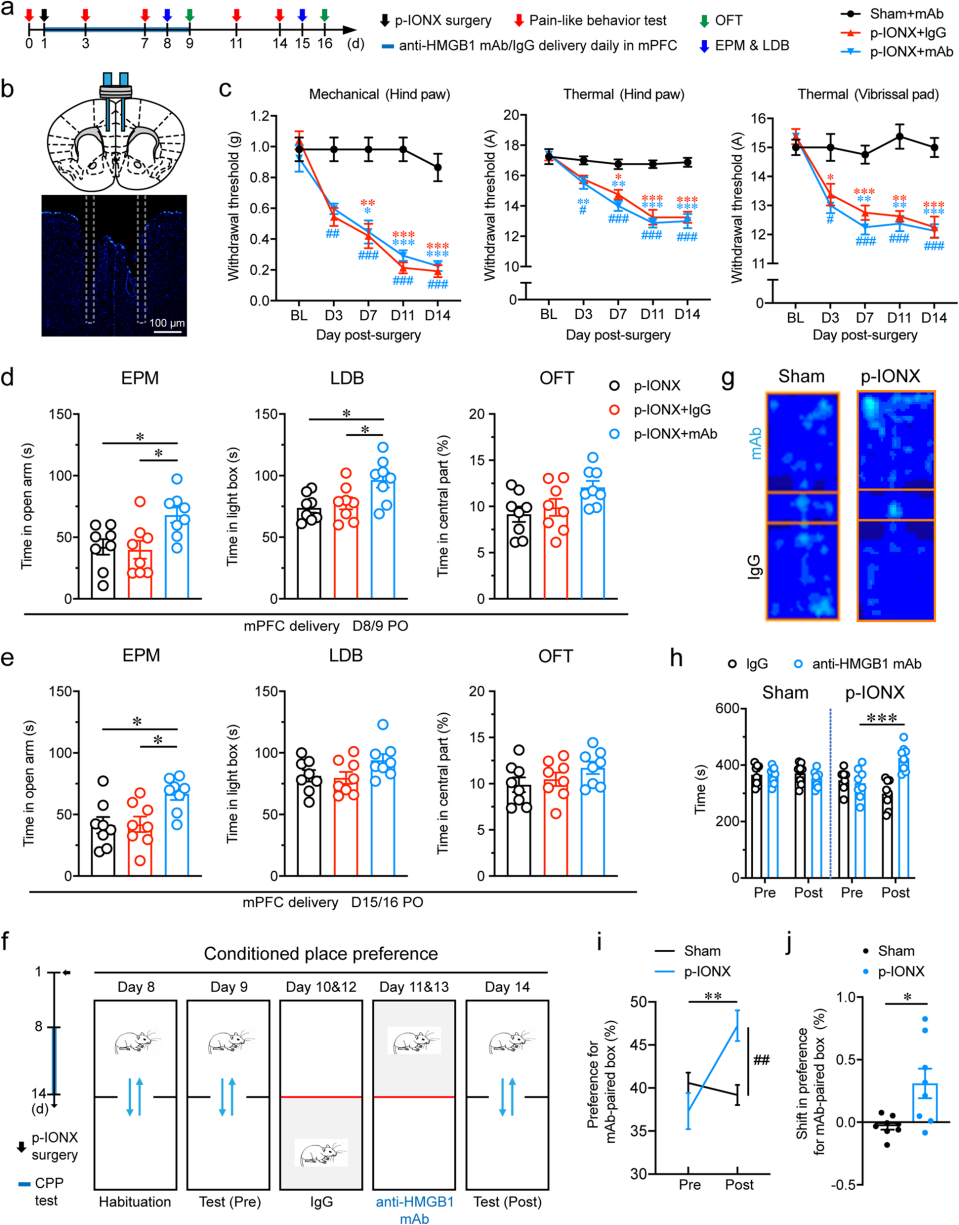
Local neutralization of HMGB1 in the mPFC reduced anxiety-like behaviors and aversion but not pain sensitization after p-IONX. **a** Schedule of procedures of mPFC drug delivery experiments in the early phase after p-IONX. **b** Schematic (upper) and representative photomicrograph (lower) of the cannula implantation in bilateral mPFC for local infusion of agents. Dashed lines indicate the trace of implanted canula. Scale bar, 100 μm. **c** Paw withdrawal thresholds to mechanical stimulation and noxious heat stimulation at the left hind paw and head withdrawal threshold to noxious heat stimulation at the left vibrissal pad before and after p-IONX. *, ** and *** indicate *P* < 0.05, 0.01 and 0.001, respectively, compared with the baseline (BL), Kruskal–Wallis test with repeated measures and Dunn’s *post hoc*. #, ## and ### indicate *P* < 0.05, 0.01 and 0.001, respectively, compared with the sham + mAb group at the same time points, two-way ANOVA with Bonferroni *post hoc*. **d, e** Anxiety-like behaviors measured by EPM, LDB and OFT on D8/9 (**d**) and D15/16 (**e**) after p-IONX, respectively. * indicates *P* < 0.05, compared with the indicated groups, ordinary one-way ANOVA with Tukey *post hoc*. **f** Schedule (left) and schematic (right) of the experiment procedures for the CPP test. **g** Representative heat maps in the CPP test. **h-j** Time spend by the mice in the control IgG- and anti-HMGB1 mAb-paired boxes (**h**), preference (**i**) and shift preference (**j**) for the anti-HMGB1 mAb-paired box in pre- (Pre) and post-conditioning (Post) phases. *, ** and *** indicate *P* < 0.05, 0.01 and 0.001, respectively, compared with the indicated groups, unpaired *t* test. ## indicates *P* < 0.01, compared with the sham group in (**i**), two-way ANOVA with Bonferroni *post hoc*. *n* = 8/group

To investigate the function of HMGB1 upregulation after p-IONX, anti-HMGB1 mAb (2 μg) was infused into bilateral mPFC for consecutive 9 days starting immediately after surgery (Fig. 4a and b). We found that the pain thresholds in the hind paw and vibrissal pad were not different between the two groups treated with anti-HMGB1 mAb and the control IgG, respectively, either during (D3 and D7 PO) or after (D11 and D14 PO) the treatment (Fig. 4c). However, this treatment alleviated anxiety-like behaviors after p-IONX on D8/9 PO (Fig. 4d). The preventive anxiolytic effect outlasted the treatment period because animals receiving anti-HMGB1 mAb still spent more time in the open arms in the EPM test until D15 PO, compared with those receiving nothing or control IgG (Fig. 4e). In addition, anti-HMGB1 mAb treatment induced place preference in the CPP test carried out one week after surgery, indicating relief of aversion (Fig. 4f-j). By contrast, local injection of anti-HMGB1 mAb into bilateral BLA where HMGB1 was also upregulated after p-IONX did not affect anxiety onset and pain sensitization (Supplementary Fig. 3). These results indicate that HMGB1 upregulation in the mPFC rather than that in the BLA is required for anxiety onset and aversion but not for pain sensitization in mice with widespread neuropathic pain.

### HMGB1 upregulation in the mPFC maintained comorbid anxiety in neuropathic pain

**Fig. 5:**
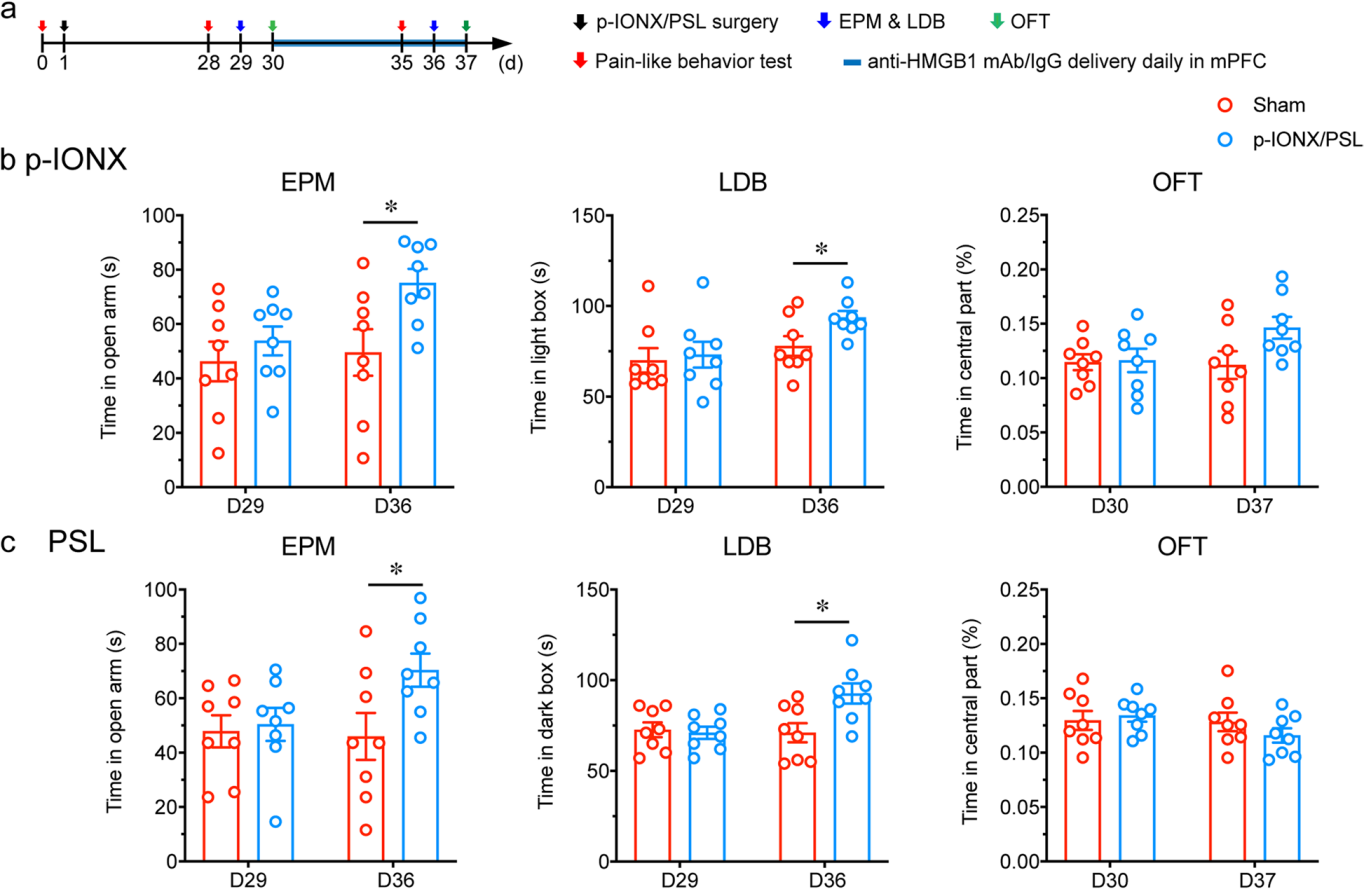
HMGB1 upregulation in the mPFC maintained comorbid anxiety in neuropathic pain. **a** Schedule of procedures of mPFC drug delivery experiments in the late phase after p-IONX. **b, c** Anxiety-like behaviors measured by EPM, LDB and OFT on D29/30 and D36/37 after p-IONX and PSL, respectively. * indicates *P* < 0.05, compared with the IgG-treated groups, unpaired *t* test. *n* = 8/group

To investigate whether HMGB1 participates in the maintenance of comorbid anxiety in neuropathic pain, anti-HMGB1 mAb (2 μg) was infused into bilateral mPFC once daily throughout D30 to D37 after p-IONX and PSL, respectively, when anxiety had been established in both groups of mice (Fig. 5a). We found that anti-HMGB1 mAb attenuated anxiety-like behaviors in mice of both models (Fig. 5b and c). These results indicate that HMGB1 in the mPFC maintains the anxiety state of mice under either widespread or somatic neuropathic pain conditions.

### HMGB1 upregulation in the mPFC was sufficient to induce anxiety and aversion but not pain sensitization

**Fig. 6:**
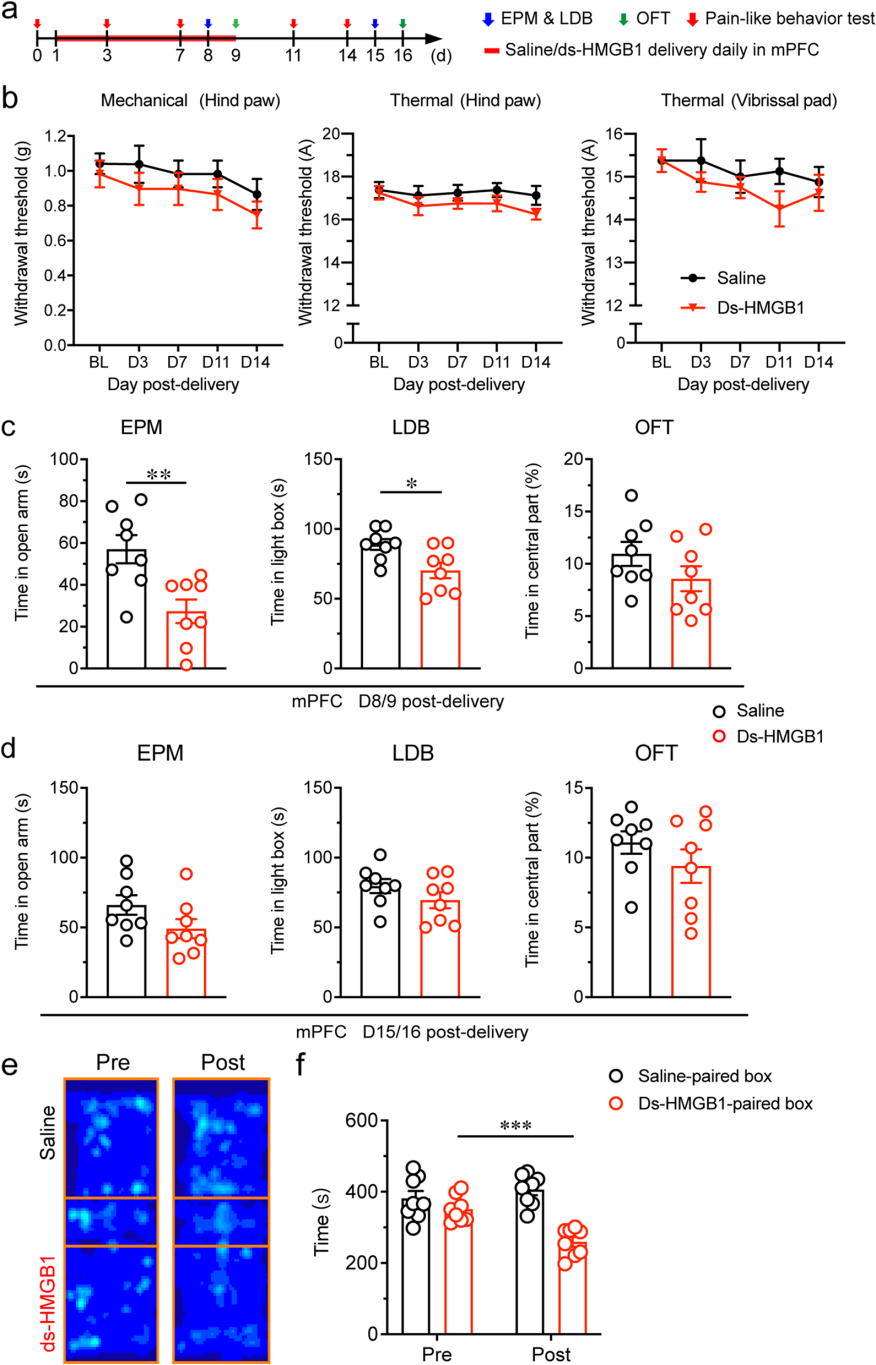
HMGB1 in the mPFC was sufficient to induce anxiety-like behaviors and aversion but not pain sensitization in naïve mice. **a** Schedule of experimental procedures. **b** Paw withdrawal thresholds to mechanical stimulation and noxious heat stimulation at the left hind paw and head withdrawal threshold to noxious heat stimulation at the left vibrissal pad. **c, d** Anxiety-like behaviors measured by EPM, LDB and OFT on D8/9 (**c**) and D15/16 (**d**) after administration. **e** Representative heat maps in the CPP test. **f** Time spend by mice in the saline- and ds-HMGB1-paired boxes. *, ** and *** indicate *P* < 0.05, 0.01 and 0.001, respectively, compared with the saline-treated group in (**c**) and pre-conditioning in (**f**), unpaired *t* test. *n* = 8/group

To further verify the contribution of mPFC HMGB1 to anxiety, ds-HMGB1 (1 μg), the pro-inflammatory form of HMGB1, was infused into bilateral mPFC of intact mice once daily for consecutive 9 days (Fig. 6a). The pain thresholds in the hind paw and vibrissal pad did not significantly change during (D3 and D7) and after (D11 and D14) the infusion (Fig. 6b). However, ds-HMGB1 infusion induced anxiety-like behaviors, which vanished one week after the cessation of infusion (Fig. 6c&d). In the CPP test, mice spent less time in the box paired with ds-HMGB1 treatment, demonstrating aversion was elicited (Fig. 6e and f). These results together indicate that HMGB1 upregulation in the mPFC is sufficient to induce anxiety and aversion, but not pain sensitization in mice.

### HMGB1 was associated with the hyperexcitability of mPFC pyramidal neurons after p-IONX

**Fig. 7:**
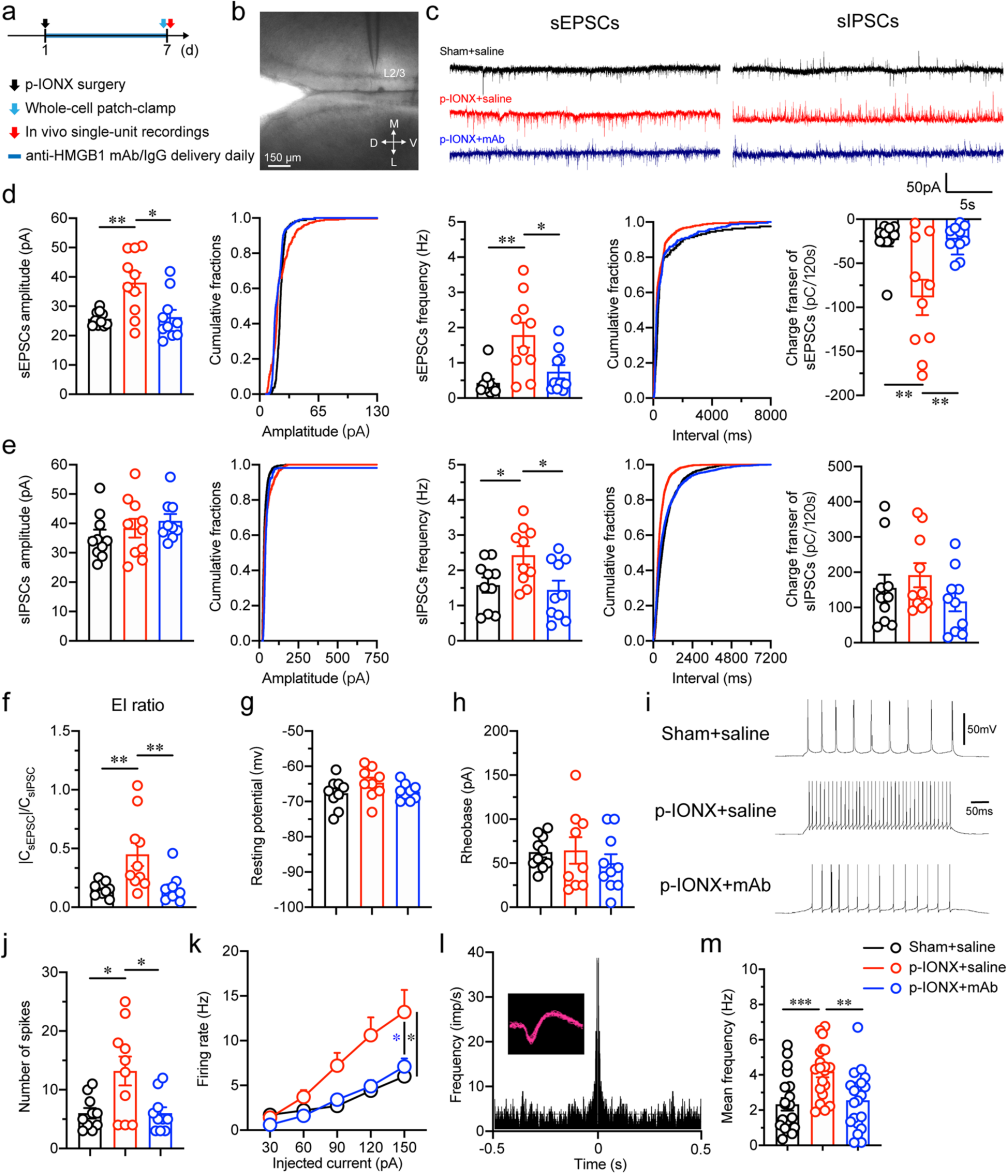
Systemic anti-HMGB1 mAb reversed the hyperexcitability of pyramidal neurons in mPFC layer 2/3 after p-IONX. **a** Schedule of experimental procedures. Anti-HMGB1 mAb was administered intraperitoneally. **b** Example image of whole cell patch-clamp recordings on mPFC layer 2/3 pyramidal neurons. Scale bar, 150 μm. **c** Example traces of sEPSCs and sIPSCs in sham + saline, p-IONX + saline and p-IONX + anti-HMGB1 mAb groups. **d, e** Amplitude, frequency and charge transfer of sEPSCs (**d**) and sIPSCs (**e**). **f** Quantification of E/I ratio (∣CsEPSC∣/CsIPSC). **g, h** Resting potential (**g**) and rheobase (the lowest current that evoked action potential firing, **h**). **i, j** Representative traces of firing (**i**) and number of spikes (**j**) of action potentials evoked by injecting current at two-fold of rheobase. **k** Firing rate of action potentials evoked by step-current injection. **l** Autocorrelation and waveform (inset) of a representative glutamatergic neuron in in vivo single-unit recordings. **m** Mean frequency of spontaneous firing. *, ** and *** indicate *P* < 0.05, 0.01 and 0.001, respectively, compared with the indicated groups, unpaired *t* test or Mann–Whitney test. *n* = 9–10 neurons from 4 mice/group in (**d**-**k**) and *n* = 20 neurons from 8 mice/group in (**m**)

It is postulated that pyramidal neurons in the mPFC are involved in regulating mood states. To investigate whether HMGB1 upregulation drives anxiety after p-IONX by influencing activities of mPFC pyramidal neurons, anti-HMGB1 mAb was intraperitoneally administered once daily immediately after p-IONX and the electrophysiological characteristics of mPFC pyramidal neurons in layer 2/3 were then evaluated on D7 PO (Fig. 7a and b). The action of systemic anti-HMGB1 mAb in the CNS was verified by the decreases in HMGB1 expression in the mPFC, BLA and medulla (Supplementary Fig. 4). We found that the amplitude and frequency of sEPSCs, but not sIPSCs, were significantly increased after p-IONX (Fig. 7c-e). The charge transfer of sEPSCs (CsEPSC) rather than that of sIPSCs (CsIPSC) was also increased, resulting in an elevation of E/I ratio (∣CsEPSC∣/CsIPSC) (Fig. 7c-f). In addition, although the resting potential and rheobase (the lowest current that evoked action potential firing) of these neurons were not changed (Fig. 7g and h), more action potentials were evoked when injecting current at two-fold of rheobase and the firing rate rose more rapidly along with the increase of current intensity by steps (Fig. 7i-k). Meanwhile, in vivo single unit recordings revealed that the spontaneous firing of these neurons was significantly increased after p-IONX (Fig. 7l and m). However, these electrophysiological changes induced by ION injury were all reversed by systemic anti-HMGB1 mAb treatment (Fig. 7c-m). By contrast, neurons in layer 5/6 showed decreases in sEPSC frequency and E/I ratio after p-IONX, on which anti-HMGB1 mAb had no effect (Supplementary Fig. 5). These results indicate that HMGB1 upregulation in the mPFC enhances excitatory synaptic transmission and excitability of pyramidal neurons in layer 2/3 after p-IONX. To further verify the causative role of hyperexcitability of mPFC pyramidal neurons in anxiety after p-IONX, these neurons were selectively inhibited by optogenetic approach (Supplementary Fig. 6a&b). Arch virus selectively targeting glutamatergic neurons was infused into the mPFC. We found that after optically inhibiting glutamatergic neurons by yellow light in the mPFC on D14 PO, although the mechanical and thermal pain thresholds in the hind paw were not changed (Supplementary Fig. 6c), anxiety was alleviated as detected by EPM and LDB tests (Supplementary Fig. 6d). These results indicate that the hyperexcitability of mPFC pyramidal neurons underlies anxiety after p-IONX.

### Systemic anti-HMGB1 mAb simultaneously alleviated pain sensitization and anxiety after p-IONX

**Fig. 8:**
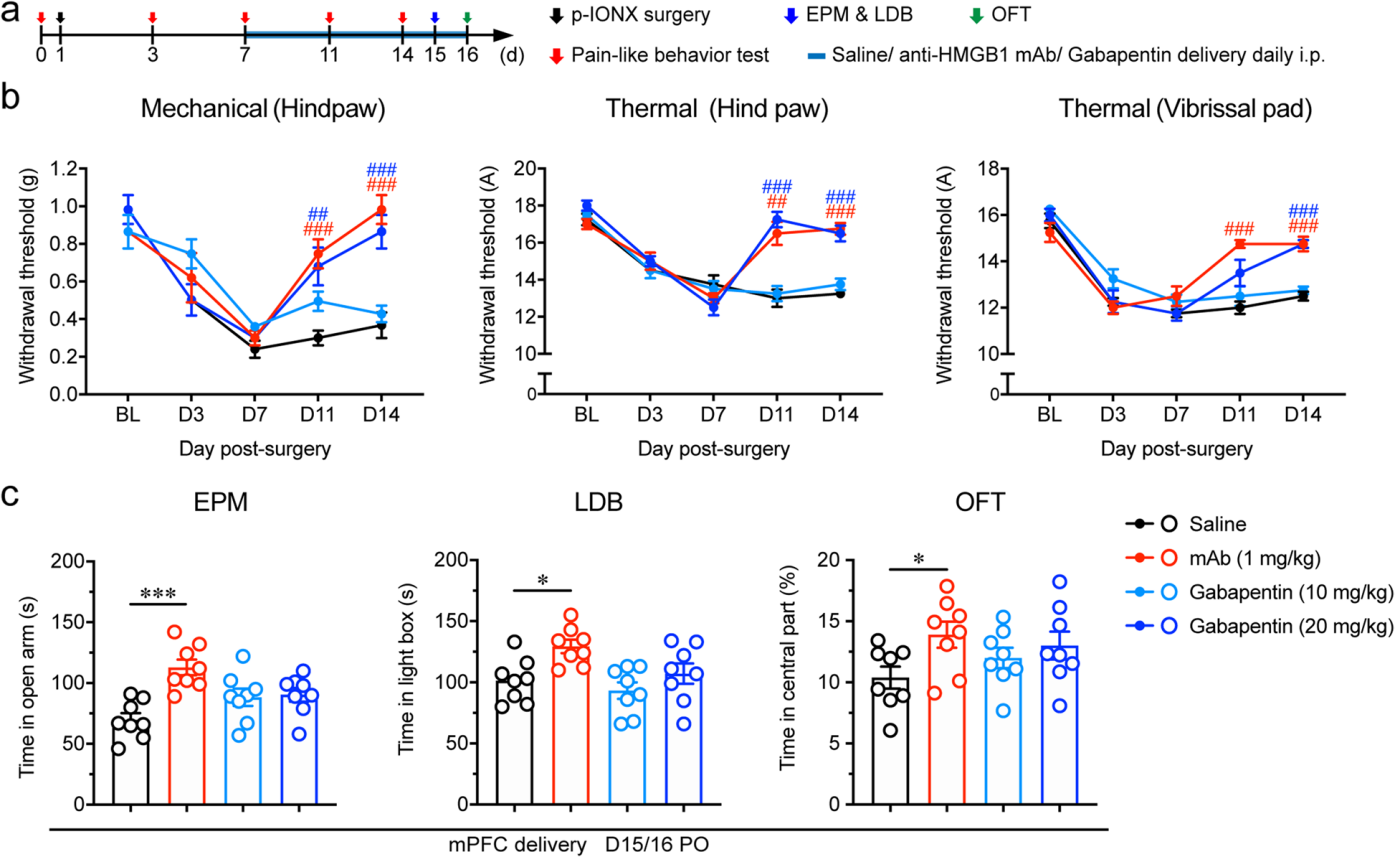
Systemic anti-HMGB1 mAb simultaneously alleviated pain sensitization and anxiety after p-IONX. **a** Schedule of experimental procedures. **b** Paw withdrawal thresholds to mechanical stimulation and noxious heat stimulation at the left hind paw and head withdrawal threshold to noxious heat stimulation at the left vibrissal pad before and after p-IONX. ## and ### indicate *P* < 0.01 and 0.001, respectively, compared with the saline-treated group at the same time points, two-way ANOVA with Bonferroni *post hoc*. **c** Anxiety-like behaviors measured by EPM, LDB and OFT on D15/16 after p-IONX. * and *** indicate *P* < 0.05 and 0.001, respectively, compared with the saline-treated group, ordinary one-way ANOVA with Dunnett *post hoc*. *n* = 8/group

Our previous studies have shown anti-HMGB1 mAb given systemically inhibits neuropathic pain and epileptic seizures, suggesting its potential as a therapeutic agent [22, 27]. Here we further examined its effect on comorbid anxiety in neuropathic pain. Anti-HMGB1 mAb (1 mg/kg) was given intraperitoneally once daily for consecutive 9 days from D7 to D16 after p-IONX when anxiety was established (Fig. 8a). Behavioral tests revealed that anti-HMGB1 mAb alleviated not only pain sensitization (Fig. 8b) but also anxiety-like behaviors (Fig. 8c). By contrast, gabapentin, one of the first line agents for the management of neuropathic pain, alleviated pain sensitization only at high (20 mg/kg), but not low (10 mg/kg) dose (Fig. 8b), while neither dose showed effect on comorbid anxiety (Fig. 8c). These results indicate that systemic administration of anti-HMGB1 mAb once daily alleviates neuropathic pain and anxiety comorbidity simultaneously.

## Discussion

Previously we have reported that p-IONX induces widespread, but PSL only induces somatic neuropathic pain [37]. Here we further found that anxiety-like behaviors developed earlier after p-IONX than PSL, consistent with previous reports that anxiety-like behaviors are exhibited earlier after ION injury than sciatic nerve injury [8–11, 38]. Clinical observations have shown that affective symptoms are positively correlated with the intensity and affected area of pain in patients [39]. Given orofacial pain, of which trigeminal pain is the most common and excruciating form, often spreads to adjacent or distant body regions [40, 41], it is very likely that the severe and widespread pain accelerates anxiety onset in mice after p-IONX. Intriguingly, no depression-like behaviors were observed up to 4 weeks in p-IONX mice. It has been reported that depressive behaviors presented at least 6 weeks after sciatic nerve injury in mice and are absent up to 45 days after trigeminal nerve injury in rats [8, 11]. These results together suggest that injuries to the orofacial and spinal nerve induce affective disorders with different characteristics and evoke anxiety more readily than depression. Because early onset anxiety per se and its deleterious impact on pain bring more suffering to patients, effective and timely management is essential. Elucidation of the mechanisms underlying early onset anxiety after p-IONX potentially provokes the development of new therapeutic strategies.

 By comparing HMGB1 expression in the brain regions related to anxiety, we found that HMGB1 upregulation in the mPFC was temporally correlated with the early and late onset anxiety after p-IONX and PSL, respectively. Taking advantage of local neutralization of HMGB1 by specific mAb, we revealed that HMGB1 in the mPFC not only drives, but also maintains anxiety-like behaviors and aversion after nerve injury. Moreover, exogenous HMGB1 locally infused into the mPFC was anxiogenic and aversive in naïve animals. These results demonstrate a causal link between HMGB1 upregulation in the mPFC and negative affect after nerve injury. It is known that after translocated into the cytoplasm and then released into the extracellular space, HMGB1 promotes inflammation via binding to several inflammatory-associated receptors [18, 19]. We found that HMGB1 was mainly secreted by neurons and this process was accompanied with the activation of microglia and astrocytes in the mPFC after p-IONX. These results together indicate that the anxiety comorbidity of neuropathic pain is associated with neuroinflammation evoked by neuron-derived HMGB1 in the mPFC. On the other hand, the absence of association of HMGB1 upregulation in the BLA with anxiety and pain sensitization indicates mild contribution of HMGB1 to these two phenotypes. The BLA was reported to be involved in controlling anxiety after sciatic nerve injury or local infusion of lipopolysaccharide [42, 43]. The discrepancies with the present study suggest that mechanisms underlying anxiety status with different etiologies are very likely different. Nonetheless, the contributions of HMGB1 upregulation in brain regions outside the mPFC including the BLA to other consequences of nerve injury, e.g. fear memory and cognition deficit, are worth of further investigation.

The dysfunction of mPFC neurons has been implicated in anxiety after various insults [44]. Here, we found that layer 2/3 pyramidal neurons were hyperactive one week after p-IONX, temporally in parallel to HMGB1 upregulation and anxiety onset. Moreover, antagonizing HMGB1 reversed the hyperexcitability of these neurons and attenuated anxiety of mice concurrently, demonstrating a predominant role of HMGB1 in these electrophysiological and behavioral changes. HMGB1 is reported to increase neuronal excitability either by promoting neuroinflammation or acting in synergy with other potent inflammatory molecules [45, 46]. Therefore, we deduced that HMGB1 in the mPFC induces anxiety rather than pain sensitization by activating neurons in layer 2/3, hence anti-HMGB1 mAb is anxiolytic but not analgesic. By contrast, excitatory neurotransmission to pyramidal neurons in layer 5/6 were suppressed after p-IONX, on which HMGB1 neutralization had no effect, indicating HMGB1 does not participate in suppressing these neurons. These results also imply that different subgroups of neurons in the mPFC respond to HMGB1 in different ways. The hypoactivity of mPFC layer 5/6 pyramidal neurons after nerve injury is proposed to contribute neuropathic pain at least by impairing the efficacy of descending pain inhibitory system [47, 48]. The absence of analgesic effect of local anti-HMGB1 mAb in the mPFC may be partly explained by the unchanged neuronal excitability. Intriguingly, optogenetic inhibition of mPFC pyramidal neurons alleviated anxiety-like behaviors but not pain sensitization after p-IONX. These findings support the causative role of hyperactive mPFC glutamatergic neurons in comorbid anxiety of neuropathic pain, but are not coincident with those reporting optogenetic manipulation of mPFC neurons results in pain modulation [49]. The discrepancy is probably due to different models used in the studies. Moreover, instant optical manipulation might be not sufficient to produce modulatory effect on neuropathic pain, as we previously reported [50].

 Although clinical studies have showed that negative affect deteriorates pain and prevents its recovery, the absence of interaction was often reported [42, 51, 52]. In addition, anxio-depressive behaviors appear weeks after nerve injury, and retain weeks after cure of neuropathic hypersensitivity [53, 54], indicating pain and anxiety are not necessarily concomitant. Here we found that attenuation of anxiety after local infusion of anti-HMGB1 mAb in the mPFC was not accompanied with pain alleviation in p-IONX mice and vice versa in mice treated with gabapentin. Coincidently, HMGB1 infusion in the mPFC induced anxiety and aversion but did not affect pain sensitivity in naïve mice. The disassociation of pain and negative affect suggests that mechanisms underlying these two consequences of nerve injury are very likely separate but with overlapping to some extent, although they may promote each other after nerve injury. Therefore, they should be treated as independent and equally important disorders. Because of the limited efficacy of current therapies for neuropathic pain and mood disorders, identification of common therapeutic targets is of great clinical significance. Considering the fact that HMGB1 in the periphery and spinal cord contributes to pain abnormalities after nerve injury [21, 23, 55, 56]. we conclude that HMGB1 is involved in both the sensory and negative affective consequences of nerve injury by acting in different sites of the nervous system and may serve as a potential therapeutic target for both pain and comorbid anxiety.

 Supportively, we found that systemic anti-HMGB1 mAb simultaneously alleviated anxiety-like behaviors and pain sensitization in p-IONX mice while reducing HMGB1 expression in the CNS. Normally, blood–brain barrier (BBB) hinders the exchange of large molecules between the blood and CNS. However, its permeability can be increased evidently under a variety of pathophysiological conditions, providing a doorway for large molecules into the CNS. It has been well recognized that peripheral nerve injury causes neuroinflammation in the CNS, which can disrupt the BBB [57, 58]. The current finding of HMGB1 downregulation in the CNS after systemic mAb was indicative of a direct intracranial effect, although the possibility of secondary reaction to peripheral neutralization cannot be completely excluded. Taken together, our results demonstrate the efficacy of systemic administration of anti-HMGB1 mAb on both neuropathic pain and anxiety comorbidity in the mouse model. The analgesic effect was comparable to that of gabapentin, which did not show anxiolytic effect. These findings in turn verify the causative roles of HMGB1 in both pain sensitization and negative affect after nerve injury and its validity and superiority as a therapeutic target.

## Conclusion

In summary, we identified HMGB1 in the mPFC as a determinant factor for the onset and maintenance of comorbid anxiety in neuropathic pain. This study not only sheds new light on the pathophysiological mechanisms of negative affect comorbidity in neuropathic pain, but also justifies targeting HMGB1 as a promising therapeutic strategy.

## Supplementary Information

**Additional file 1:** **Supplementary Fig. 1** p-IONX induced widespread neuropathic pain, anxiety- like behaviors and HMGB1 upregulation in the mPFC of C57BL/6J mice.** a **Schedule of experimental procedures. **b **Paw withdrawal thresholds to mechanical stimulation and noxious heat stimulation at the left hind paw and head withdrawal thresholds to noxious heat stimulation at the left vibrissal pad, respectively. *, ** and *** indicate *P *< 0.05, 0.01 and 0.001, respectively, compared with baseline (BL), Kruskal-Wallis test with repeated measures and Dunn’s *post hoc*. ## and ### indicate *P *< 0.01 and 0.001, respectively, compared with the sham group at the same time points, two-way ANOVA with Bonferroni *post hoc*. *n *= 8/group. **c **Anxiety-like behaviors measured by EPM, LDB and OFT tests. *, ** and *** indicate *P *< 0.05, 0.01 and 0.001, respectively, compared with the sham group, unpaired *t* test. *n *= 8/group. **d **Representative photomicrographs of HMGB1 immunostaining in the mPFC indicated by the red box in the left panel on D16 PO. Scale bar, 200 μm. **e **Quantification of HMGB1 fluorescence intensity in the mPFC on D9 and D16 PO. ** indicates *P *< 0.01, compared with the sham group, unpaired *t* test. *n *= 4/group. **Supplementary Fig. 2** HMGB1 expression was upregulated in multiple brain regions after p-IONX in MRL/MPJ mice.** a **Schematic of experimental procedures. **b **Representative photomicrographs of HMGB1 immunostaining in the cortex, thalamus and amygdala. AI, agranular insular cortex; Au1, primary auditory cortex; AuV, secondary auditory cortex, ventral area; BLP, basolateral amygdaloid nucleus, posterior part; BMP, basomedial amygdaloid nucleus, posterior part; M1, primary motor cortex; MD, mediodorsal thalamic nucleus; PVA, paraventricular thalamic nucleus, anterior part; S1, primary sensory cortex; TeA, temporal association cortex; VPL, ventral posterolateral thalamic nucleus; VPM, ventral posteromedial thalamic nucleus. Scale bar, 200 μm. **Supplementary Fig. 3** HMGB1 upregulation in the BLA did not affect anxiety onset and pain sensitization after p-IONX in MRL/MPJ mice.** a **Schedule of procedures of BLA drug delivery experiments. **b **Schematic of canula implantation in bilateral BLA (upper) and representative photomicrograph in the left BLA (lower). Dashed lines indicate the trace of implanted canula. Scale bar, 100 μm. **c **Paw withdrawal thresholds to mechanical stimulation and noxious heat stimulation at the left hind paw and head withdrawal threshold to noxious heat stimulation at the ipsilateral vibrissal pad before and after p-IONX. ** and *** indicate *P *< 0.01 and 0.001, respectively, compared with the baseline (BL), Kruskal-Wallis test with repeated measures and Dunn’s *post hoc*. #, ## and ### indicate *P *< 0.05, 0.01 and 0.001, respectively, compared with the sham + mAb group at the same time points, two-way ANOVA with Bonferroni *post hoc*. **d **Anxiety-like behaviors measured by EPM, LDB and OFT tests on D8/9 after p-IONX. *n *= 8/group. **Supplementary Fig. 4 **Systemic anti-HMGB1 mAb reduced HMGB1 expression after p-IONX in MRL/MPJ mice.** a **Schedule of experimental procedures. **b-c **Representative images of protein bands in western blotting (**b**) and quantification of HMGB1 (**c**) in the mPFC, BLA and medulla on D7 after p-IONX. * and ** indicate *P *< 0.05 and 0.01, respectively, compared with the indicated groups, unpaired *t* test. *n *= 4/group. **Supplementary Fig. 5 **Systemic anti-HMGB1 mAb did not affect the reduced activity of pyramidal neurons in mPFC layer 5/6 after p-IONX in MRL/MPJ mice.** a **Schedule of experimental procedures. **b **Example of sEPSCs and sIPSCs in sham + saline, p-IONX+ saline and p-IONX+ anti-HMGB1 mAb groups. **c-d **Amplitude, frequency and charge transfer of sEPSCs (**c**) and sIPSCs (**d**). **e **Quantification of E/I ratio. **f **Resting potential. **g **Rheobase. **h-i **Representative traces of action potential firing (**h**) and number of spikes (**i**) evoked by injecting current at two-fold of rheobase. * and ** indicate *P < *0.05 and 0.01, respectively, compared with the indicated groups, unpaired *t* test or Mann-Whitney test. *n *= 9-10 neurons from 4 mice/group. **Supplementary Fig. 6 **Optogenetic inhibition of mPFC pyramidal neurons alleviated anxiety after p-IONX. a Schematic drawing of sites for virus injection and optical fiber placement. b Example photomicrographs showing the Arch-eYFP in mPFC (left) and colocalization of eYFP and CaMKIIα, a marker of glutamatergic neurons (right). **c** Example neuron in the mPFC that deceased its firing frequency in response to yellow laser stimulation. **d** Paw withdrawal thresholds to mechanical stimulation and noxious heat stimulation. **e** Anxiety-like behaviors measured by OFT and EPM tests on D14 after surgery. ** indicates *P <* 0.01, compared with the laser off group, unpaired *t* test. *n* = 8-10/group.

## Data Availability

The datasets used and analyzed during the current study are available from the corresponding author on reasonable request.

## Abbreviations

ACSF: Artificial cerebral spinal fluid
ANOVA: Analysis of variance
BBB: Blood–brain barrier
BLA: Basal lateral amygdala
BSCB: Blood-spinal cord barrier
CPP: Conditioned place preference
EPM: Elevated plus maze
FST: Forced swimming test
HMGB1: High mobility group box 1
ION: Infraorbital nerve
i.p.: Intraperitoneally
LDB: Light dark box
mAb: Monoclonal antibody
mPFC: Medial prefrontal cortex
OFT: Open field test
PBN: Parabrachial nucleus
p-IONX: Partial infraorbital nerve transection
PO: Postoperatively
PSL: Partial sciatic nerve ligation
sEPSC: Spontaneous excitatory postsynaptic currents
sIPSC: Spontaneous inhibitory postsynaptic currents
TST: Tail suspension test
vHPC: Ventral hippocampus

## References

[1] Failde I, Dueñas M, Ribera MV, Gálvez R, Mico JA, Salazar A, de Sola H, Pérez C (2018). Prevalence of central and peripheral neuropathic pain in patients attending pain clinics in Spain: factors related to intensity of pain and quality of life. J Pain Res.

[2] Zakrzewska JM, Wu J, Mon-Williams M, Phillips N, Pavitt SH (2017). Evaluating the impact of trigeminal neuralgia. Pain.

[3] Zhuo M (2016). Neural mechanisms underlying anxiety-chronic pain interactions. Trends Neurosci.

[4] Tang NKY, Salkovskis PM, Hodges A, Wright KJ, Hanna M, Hester J (2008). Effects of mood on pain responses and pain tolerance: an experimental study in chronic back pain patients. Pain.

[5] Gilron I, Baron R, Jensen T (2015). Neuropathic pain: principles of diagnosis and treatment. Mayo Clin Proc.

[6] Torta R, Ieraci V, Zizzi F (2017). A Review of the emotional aspects of neuropathic pain: from comorbidity to co-pathogenesis. Pain Ther.

[7] Caruso R, Ostuzzi G, Turrini G, Ballette F, Recla E, Dall'Olio R, Croce E, Casoni B, Grassi L, Barbui C (2019). Beyond pain: can antidepressants improve depressive symptoms and quality of life in patients with neuropathic pain?. A syst rev and meta-analysis Pain.

[8] Yalcin I, Bohren Y, Waltisperger E, Sage-Ciocca D, Yin JC, Freund-Mercier MJ, Barrot M (2011). A time-dependent history of mood disorders in a murine model of neuropathic pain. Biol Psychiatry.

[9] Yalcin I, Barthas F, Barrot M (2014). Emotional consequences of neuropathic pain: insight from preclinical studies. Neurosci Biobehav Rev.

[10] Cui WQ, Zhang WW, Chen T, Li Q, Xu F, Mao-Ying QL, Mi WL, Wang YQ, Chu YX (2020). Tacr3 in the lateral habenula differentially regulates orofacial allodynia and anxiety-like behaviors in a mouse model of trigeminal neuralgia. Acta Neuropathol Commun.

[11] Gambeta E, Batista MA, Maschio GP, Turnes JM, Araya EI, Chichorro JG (2018). Anxiety- but not depressive-like behaviors are related to facial hyperalgesia in a model of trigeminal neuropathic pain in rats. Physiol Behav.

[12] Maes M, Song C, Lin A, De Jongh R, Van Gastel A, Kenis G, Bosmans E, De Meester I, Benoy I, Neels H (1998). The effects of psychological stress on humans: increased production of pro-inflammatory cytokines and a Th1-like response in stress-induced anxiety. Cytokine.

[13] Rooney S, Sah A, Unger MS, Kharitonova M, Sartori SB, Schwarzer C, Aigner L, Kettenmann H, Wolf SA, Singewald N (2020). Neuroinflammatory alterations in trait anxiety: modulatory effects of minocycline. Transl Psychiatry.

[14] Padilla-Coreano N, Bolkan SS, Pierce GM, Blackman DR, Hardin WD, Garcia-Garcia AL, Spellman TJ, Gordon JA (2016). Direct Ventral Hippocampal-Prefrontal Input Is Required for Anxiety-Related Neural Activity and Behavior. Neuron.

[15] Adhikari A, Lerner TN, Finkelstein J, Pak S, Jennings JH, Davidson TJ, Ferenczi E, Gunaydin LA, Mirzabekov JJ, Ye L (2015). Basomedial amygdala mediates top-down control of anxiety and fear. Nat.

[16] Tsung A, Tohme S, Billiar TR (2014). High-mobility group box-1 in sterile inflammation. J Intern Med.

[17] Sun Y, Chen H, Dai J, Zou H, Gao M, Wu H, Ming B, Lai L, Xiao Y, Xiong P (2015). HMGB1 expression patterns during the progression of experimental autoimmune encephalomyelitis. J Neuroimmunol.

[18] Lotze MT, Tracey KJ (2005). High-mobility group box 1 protein (HMGB1): nuclear weapon in the immune arsenal. Nat Rev Immunol.

[19] Bertheloot D, Latz E (2017). HMGB1, IL-1α, IL-33 and S100 proteins: dual-function alarmins. Cell Mol Immunol.

[20] Zhang SH, Yu J, Lou GD, Tang YY, Wang RR, Hou WW, Chen Z (2016). Widespread pain sensitization after partial infraorbital nerve transection in MRL/MPJ mice. Pain.

[21] Hu TT, Yu J, Liu K, Du Y, Qu FH, Guo F, Yu LN, Nishibori M, Chen Z, Zhang SH (2020). A crucial role of HMGB1 in orofacial and widespread pain sensitization following partial infraorbital nerve transection. Brain Behav Immun.

[22] Franklin TC, Xu C, Duman RS (2018). Depression and sterile inflammation: Essential role of danger associated molecular patterns. Brain Behav Immun.

[23] Hisaoka-Nakashima K, Tomimura Y, Yoshii T, Ohata K, Takada N, Zhang FF, Nakamura Y, Liu K, Wake H, Nishibori M (2019). High-mobility group box 1-mediated microglial activation induces anxiodepressive-like behaviors in mice with neuropathic pain. Prog Neuropsychopharmacol Biol Psychiatry.

[24] Zimmermann M (1983). Ethical guidelines for investigations of experimental pain in conscious animals. Pain.

[25] Seltzer Z, Dubner R, Shir Y (1990). A novel behavioral model of neuropathic pain disorders produced in rats by partial sciatic nerve injury. Pain.

[26] Rodgers RJ, Dalvi A (1997). Anxiety, defence and the elevated plus-maze. Neurosci Biobehav Rev.

[27] Bourin M, Hascoët M (2003). The mouse light/dark box test. Eur J Pharmacol.

[28] Choleris E, Thomas AW, Kavaliers M, Prato FS (2001). A detailed ethological analysis of the mouse open field test: effects of diazepam, chlordiazepoxide and an extremely low frequency pulsed magnetic field. Neurosci Biobehav Rev.

[29] Zheng P, Zeng B, Zhou C, Liu M, Fang Z, Xu X, Zeng L, Chen J, Fan S, Du X (2016). Gut microbiome remodeling induces depressive-like behaviors through a pathway mediated by the host's metabolism. Mol Psychiatry.

[30] Cryan JF, Mombereau C, Vassout A (2005). The tail suspension test as a model for assessing antidepressant activity: review of pharmacological and genetic studies in mice. Neurosci Biobehav Rev.

[31] Bonin RP, Bories C, De Koninck Y (2014). A simplified up-down method (SUDO) for measuring mechanical nociception in rodents using von frey filaments. Mol Pain.

[32] King T, Vera-Portocarrero L, Gutierrez T, Vanderah TW, Dussor G, Lai J, Fields HL, Porreca F (2009). Unmasking the tonic-aversive state in neuropathic pain. Nat Neurosci.

[33] Liu K, Mori S, Takahashi HK, Tomono Y, Wake H, Kanke T, Sato Y, Hiraga N, Adachi N, Yoshino T, Nishibori M (2007). Anti-high mobility group box 1 monoclonal antibody ameliorates brain infarction induced by transient ischemia in rats. Faseb j.

[34] Wang Y, Xu C, Xu Z, Ji C, Liang J, Wang Y, Chen B, Wu X, Gao F, Wang S (2017). Depolarized GABAergic signaling in subicular microcircuits mediates generalized seizure in temporal lobe epilepsy. Neuron.

[35] Le Van QM, Bragin A, Staba R, Crépon B, Wilson CL, Engel J (2008). Cell type-specific firing during ripple oscillations in the hippocampal formation of humans. J Neurosci.

[36] Csicsvari J, Hirase H, Czurkó A, Mamiya A, Buzsáki G (1999). Oscillatory coupling of hippocampal pyramidal cells and interneurons in the behaving Rat. J Neurosci.

[37] Hu TT, Wang RR, Tang YY, Wu YX, Yu J, Hou WW, Lou GD, Zhou YD, Zhang SH, Chen Z (2018). TLR4 deficiency abrogated widespread tactile allodynia, but not widespread thermal hyperalgesia and trigeminal neuropathic pain after partial infraorbital nerve transection. Pain.

[38] Sheng HY, Lv SS, Cai YQ, Shi W, Lin W, Liu TT, Lv N, Cao H, Zhang L, Zhang YQ (2020). Activation of ventrolateral orbital cortex improves mouse neuropathic pain-induced anxiodepression. JCI Insight.

[39] Gerrits M, van Oppen P, van Marwijk HWJ, Penninx B, van der Horst HE (2014). Pain and the onset of depressive and anxiety disorders. Pain.

[40] Türp JC, Kowalski CJ, O'Leary N, Stohler CS (1998). Pain maps from facial pain patients indicate a broad pain geography. J Dent Res.

[41] Gambeta E, Chichorro JG, Zamponi GW (2020). Trigeminal neuralgia: An overview from pathophysiology to pharmacological treatments. Mol Pain.

[42] Llorca-Torralba M, Suárez-Pereira I, Bravo L, Camarena-Delgado C, Garcia-Partida JA, Mico JA, Berrocoso E (2019). Chemogenetic silencing of the locus coeruleus-basolateral amygdala pathway abolishes pain-induced anxiety and enhanced aversive learning in rats. Biol Psychiatry.

[43] Zheng ZH, Tu JL, Li XH, Hua Q, Liu WZ, Liu Y, Pan BX, Hu P, Zhang WH (2021). Neuroinflammation induces anxiety- and depressive-like behavior by modulating neuronal plasticity in the basolateral amygdala. Brain Behav Immun.

[44] Xu P, Chen A, Li Y, Xing X, Lu H (2019). Medial prefrontal cortex in neurological diseases. Physiol Genomics.

[45] Agalave NM, Svensson CI (2015). Extracellular high-mobility group box 1 protein (HMGB1) as a mediator of persistent pain. Mol Med.

[46] Andersson U, Yang H, Harris H (2018). High-mobility group box 1 protein (HMGB1) operates as an alarmin outside as well as inside cells. Semin Immunol.

[47] Huang J, Gadotti VM, Chen L, Souza IA, Huang S, Wang D, Ramakrishnan C, Deisseroth K, Zhang Z, Zamponi GW (2019). A neuronal circuit for activating descending modulation of neuropathic pain. Nat Neurosci.

[48] Zhang Z, Gadotti VM, Chen L, Souza IA, Stemkowski PL, Zamponi GW (2015). Role of prelimbic GABAergic circuits in sensory and emotional aspects of neuropathic pain. Cell Rep.

[49] Ong WY, Stohler CS, Herr DR (2019). Role of the prefrontal cortex in pain processing. Mol Neurobiol.

[50] Hu TT, Wang RR, Du Y, Guo F, Wu YX, Wang Y, Wang S, Li XY, Zhang SH, Chen Z (2019). Activation of the intrinsic pain inhibitory circuit from the midcingulate Cg2 to zona incerta alleviates neuropathic pain. J Neurosci.

[51] Jensen KB, Petzke F, Carville S, Fransson P, Marcus H, Williams SC, Choy E, Mainguy Y, Gracely R, Ingvar M, Kosek E (2010). Anxiety and depressive symptoms in fibromyalgia are related to poor perception of health but not to pain sensitivity or cerebral processing of pain. Arthritis Rheum.

[52] Gonzalez-Soler EM, Blasco-Serra A, Alfosea-Cuadrado GM, Igual-Lopez M, Orduna-Valls J, Tornero-Tornero C, Valverde-Navarro AA (2020). Chronic pregabalin treatment ameliorates pain, but not depressive-like behaviors, in a reserpine-induced myalgia model in rats. Pain Physician.

[53] Dimitrov EL, Tsuda MC, Cameron HA, Usdin TB (2014). Anxiety- and depression-like behavior and impaired neurogenesis evoked by peripheral neuropathy persist following resolution of prolonged tactile hypersensitivity. J Neurosci.

[54] Sellmeijer J, Mathis V, Hugel S, Li XH, Song Q, Chen QY, Barthas F, Lutz PE, Karatas M, Luthi A (2018). Hyperactivity of anterior cingulate cortex areas 24a/24b drives chronic pain-induced anxiodepressive-like consequences. J Neurosci.

[55] Nakamura Y, Morioka N, Abe H, Zhang FF, Hisaoka-Nakashima K, Liu K, Nishibori M, Nakata Y (2013). Neuropathic pain in rats with a partial sciatic nerve ligation is alleviated by intravenous injection of monoclonal antibody to high mobility group box-1. Plos one.

[56] Lin TB, Hsieh MC, Lai CY, Cheng JK, Wang HH, Chau YP, Chen GD, Peng HY (2016). Melatonin relieves neuropathic allodynia through spinal MT2-enhanced PP2Ac and downstream HDAC4 shuttling-dependent epigenetic modification of hmgb1 transcription. J Pineal Res.

[57] Beggs S, Liu XJ, Kwan C, Salter MW (2010). Peripheral nerve injury and TRPV1-expressing primary afferent C-fibers cause opening of the blood-brain barrier. Mol Pain.

[58] Nishibori M, Wang D, Ousaka D, Wake H (2020). High mobility group box-1 and blood-brain barrier disruption. Cells.

