# Supplementary material for: HMGB1 in the mPFC governs comorbid anxiety in neuropathic pain
Source: J Headache Pain. 2022 Aug 16;23(1):102. doi: 10.1186/s10194-022-01475-z (PMC9382735; doi:10.1186/s10194-022-01475-z)
Supplement: Supplementary file 1 — Additional file 1: Supplementary Fig. 1 p-IONX induced widespread neuropathic pain, anxiety- like behaviors and HMGB1 upregulation in the mPFC of C57BL/6J mice. a Schedule of experimental procedures. b Paw withdrawal thresholds to mechanical stimulation and noxious heat stimulation at the left hind paw and head withdrawal thresholds to noxious heat stimulation at the left vibrissal pad, respectively. *, ** and *** indicate P < 0.05, 0.01 and 0.001, respectively, compared with baseline (BL), Kruskal-Wallis test with repeated measures and Dunn’s post hoc. ## and ### indicate P < 0.01 and 0.001, respectively, compared with the sham group at the same time points, two-way ANOVA with Bonferroni post hoc. n = 8/group. c Anxiety-like behaviors measured by EPM, LDB and OFT tests. *, ** and *** indicate P < 0.05, 0.01 and 0.001, respectively, compared with the sham group, unpaired t test. n = 8/group. d Representative photomicrographs of HMGB1 immunostaining in the mPFC indicated by the red box in the left panel on D16 PO. Scale bar, 200 μm. e Quantification of HMGB1 fluorescence intensity in the mPFC on D9 and D16 PO. ** indicates P < 0.01, compared with the sham group, unpaired t test. n = 4/group. Supplementary Fig. 2 HMGB1 expression was upregulated in multiple brain regions after p-IONX in MRL/MPJ mice. a Schematic of experimental procedures. b Representative photomicrographs of HMGB1 immunostaining in the cortex, thalamus and amygdala. AI, agranular insular cortex; Au1, primary auditory cortex; AuV, secondary auditory cortex, ventral area; BLP, basolateral amygdaloid nucleus, posterior part; BMP, basomedial amygdaloid nucleus, posterior part; M1, primary motor cortex; MD, mediodorsal thalamic nucleus; PVA, paraventricular thalamic nucleus, anterior part; S1, primary sensory cortex; TeA, temporal association cortex; VPL, ventral posterolateral thalamic nucleus; VPM, ventral posteromedial thalamic nucleus. Scale bar, 200 μm. Supplementary Fig. 3 HMGB1 upregulation in the [file 10194_2022_1475_MOESM1_ESM.docx]

**LEGENDS FOR SUPPLEMENTARY FIGURES**


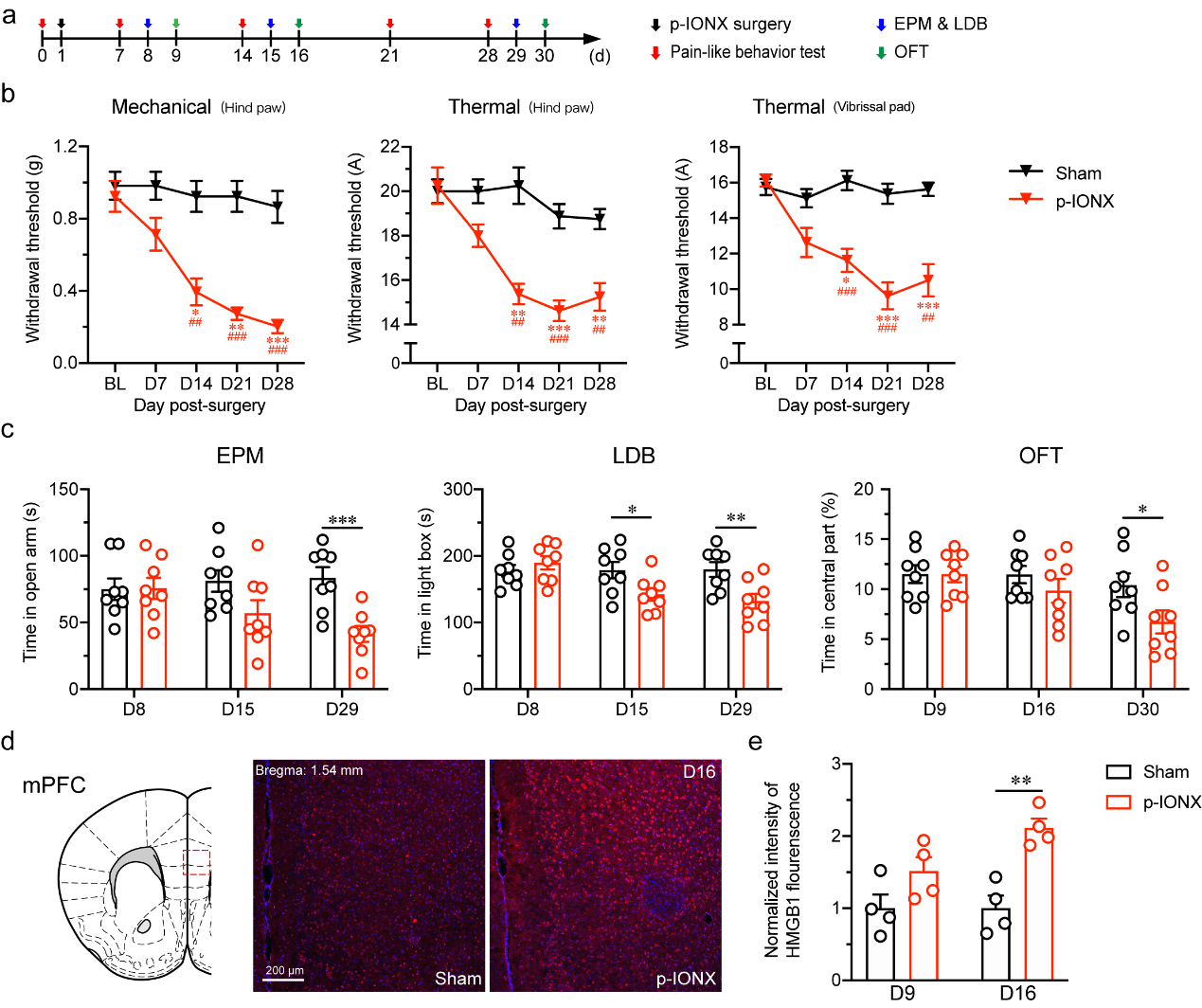


**Supplementary Fig. 1 p-IONX induced widespread neuropathic pain, anxiety- like behaviors and HMGB1 upregulation in the mPFC of C57BL/6J mice. a** Schedule of experimental procedures. **b** Paw withdrawal thresholds to mechanical stimulation and noxious heat stimulation at the left hind paw and head withdrawal thresholds to noxious heat stimulation at the left vibrissal pad, respectively. *, ** and *** indicate *P* < 0.05, 0.01 and 0.001, respectively, compared with baseline (BL), Kruskal-Wallis test with repeated measures and Dunn’s *post hoc*. ## and ### indicate *P* < 0.01 and 0.001, respectively, compared with the sham group at the same time points, two-way ANOVA with Bonferroni *post hoc*. *n* = 8/group. **c** Anxiety-like behaviors measured by EPM, LDB and OFT tests. *, ** and *** indicate *P* < 0.05, 0.01 and 0.001, respectively, compared with the sham group, unpaired *t* test. *n* = 8/group. **d** Representative photomicrographs of HMGB1 immunostaining in the mPFC indicated by the red box in the left panel on D16 PO. Scale bar, 200 μm. **e** Quantification of HMGB1 fluorescence intensity in the mPFC on D9 and D16 PO. ** indicates *P* < 0.01, compared with the sham group, unpaired *t* test. *n* = 4/group.


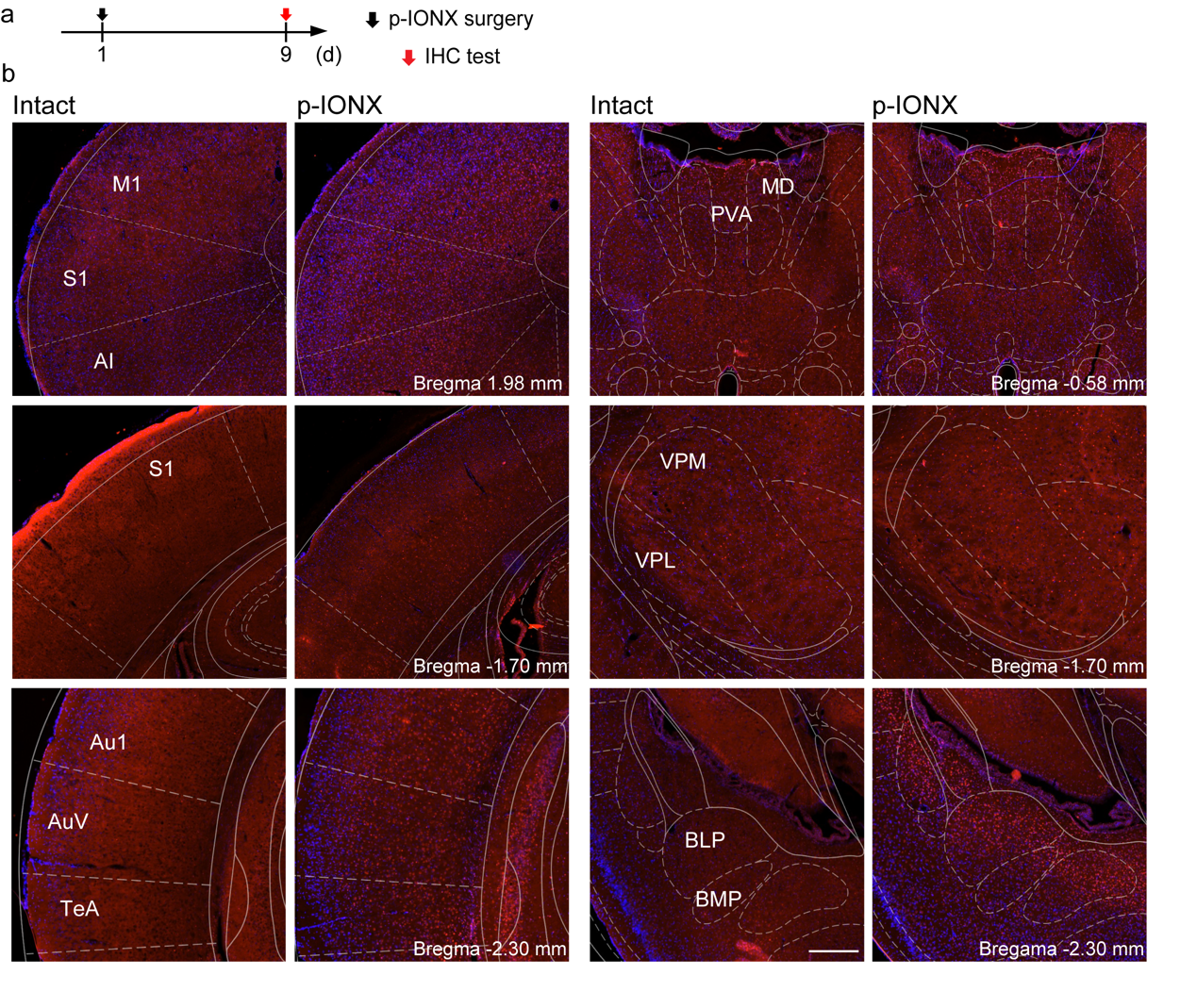


**Supplementary Fig. 2 HMGB1 expression was upregulated in multiple brain regions** **after p-IONX in MRL/MPJ mice. a** Schematic of experimental procedures. **b** Representative photomicrographs of HMGB1 immunostaining in the cortex, thalamus and amygdala. AI, agranular insular cortex; Au1, primary auditory cortex; AuV, secondary auditory cortex, ventral area; BLP, basolateral amygdaloid nucleus,

posterior part; BMP, basomedial amygdaloid nucleus, posterior part; M1, primary motor cortex; MD, mediodorsal thalamic nucleus; PVA, paraventricular thalamic nucleus, anterior part; S1, primary sensory cortex; TeA, temporal association cortex; VPL, ventral posterolateral thalamic nucleus; VPM, ventral posteromedial thalamic nucleus. Scale bar, 200 μm.

**
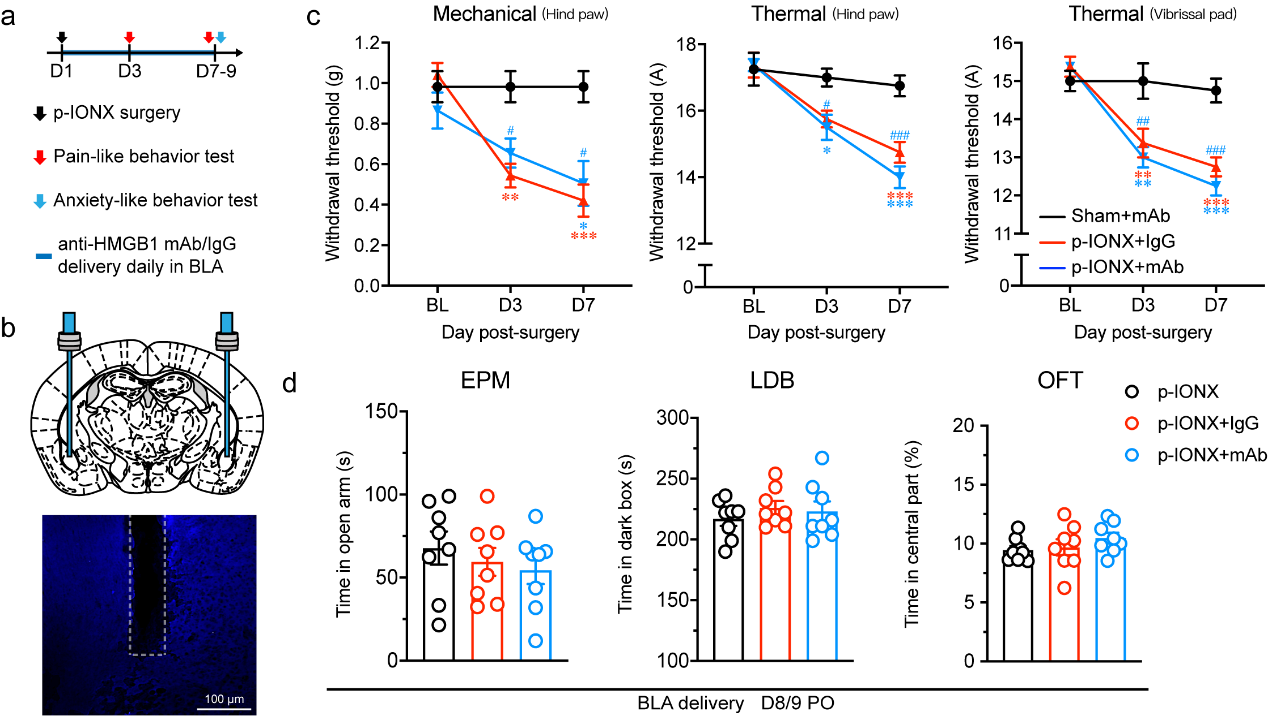
**

**Supplementary Fig. 3 HMGB1 upregulation in the BLA did not affect anxiety onset and pain sensitization after p-IONX in MRL/MPJ mice. a** Schedule of procedures of BLA drug delivery experiments. **b** Schematic of canula implantation in bilateral BLA (upper) and representative photomicrograph in the left BLA (lower). Dashed lines indicate the trace of implanted canula. Scale bar, 100 μm. **c** Paw withdrawal thresholds to mechanical stimulation and noxious heat stimulation at the left hind paw and head withdrawal threshold to noxious heat stimulation at the ipsilateral vibrissal pad before and after p-IONX. ** and *** indicate *P* < 0.01 and 0.001, respectively, compared with the baseline (BL), Kruskal-Wallis test with repeated measures and Dunn’s *post hoc*. #, ## and ### indicate *P* < 0.05, 0.01 and 0.001, respectively, compared with the sham + mAb group at the same time points, two-way ANOVA with Bonferroni *post hoc*. **d** Anxiety-like behaviors measured by EPM, LDB and OFT tests on D8/9 after p-IONX. *n* = 8/group.


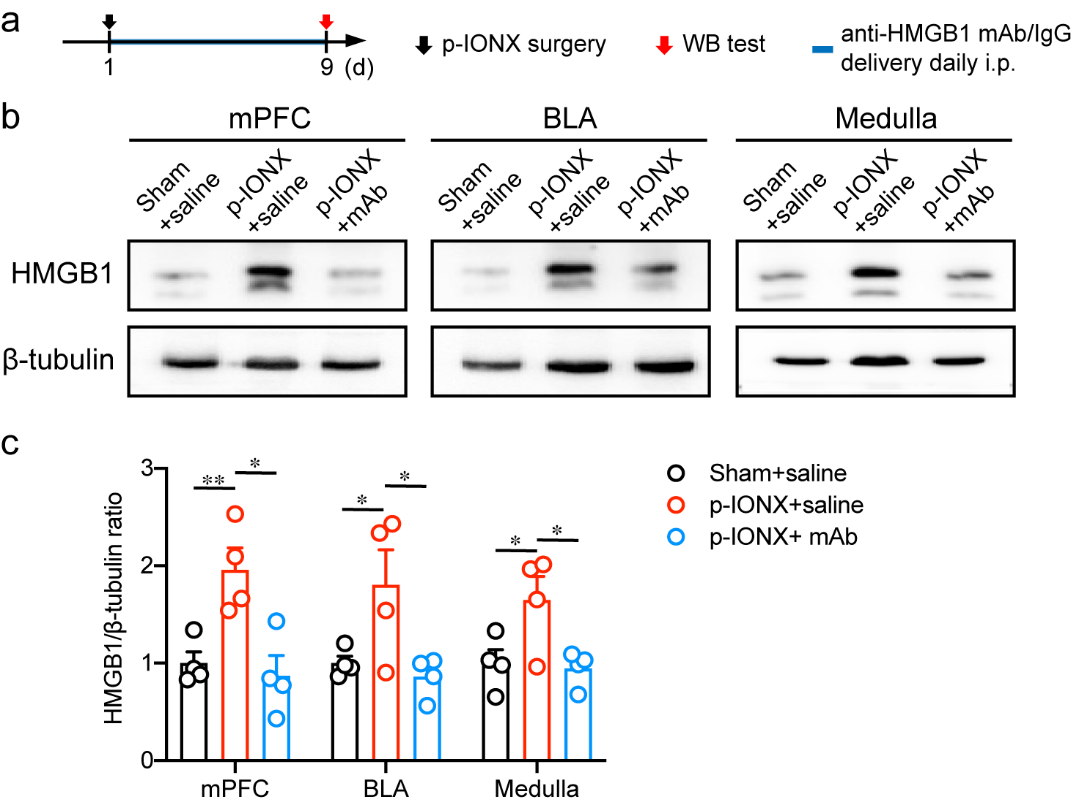


**Supplementary Fig. 4 Systemic anti-HMGB1 mAb reduced HMGB1 expression after p-IONX in MRL/MPJ mice. a** Schedule of experimental procedures. **b-c** Representative images of protein bands in western blotting (**b**) and quantification of HMGB1 (**c**) in the mPFC, BLA and medulla on D7 after p-IONX. * and ** indicate *P* < 0.05 and 0.01, respectively, compared with the indicated groups, unpaired *t* test. *n* = 4/group.


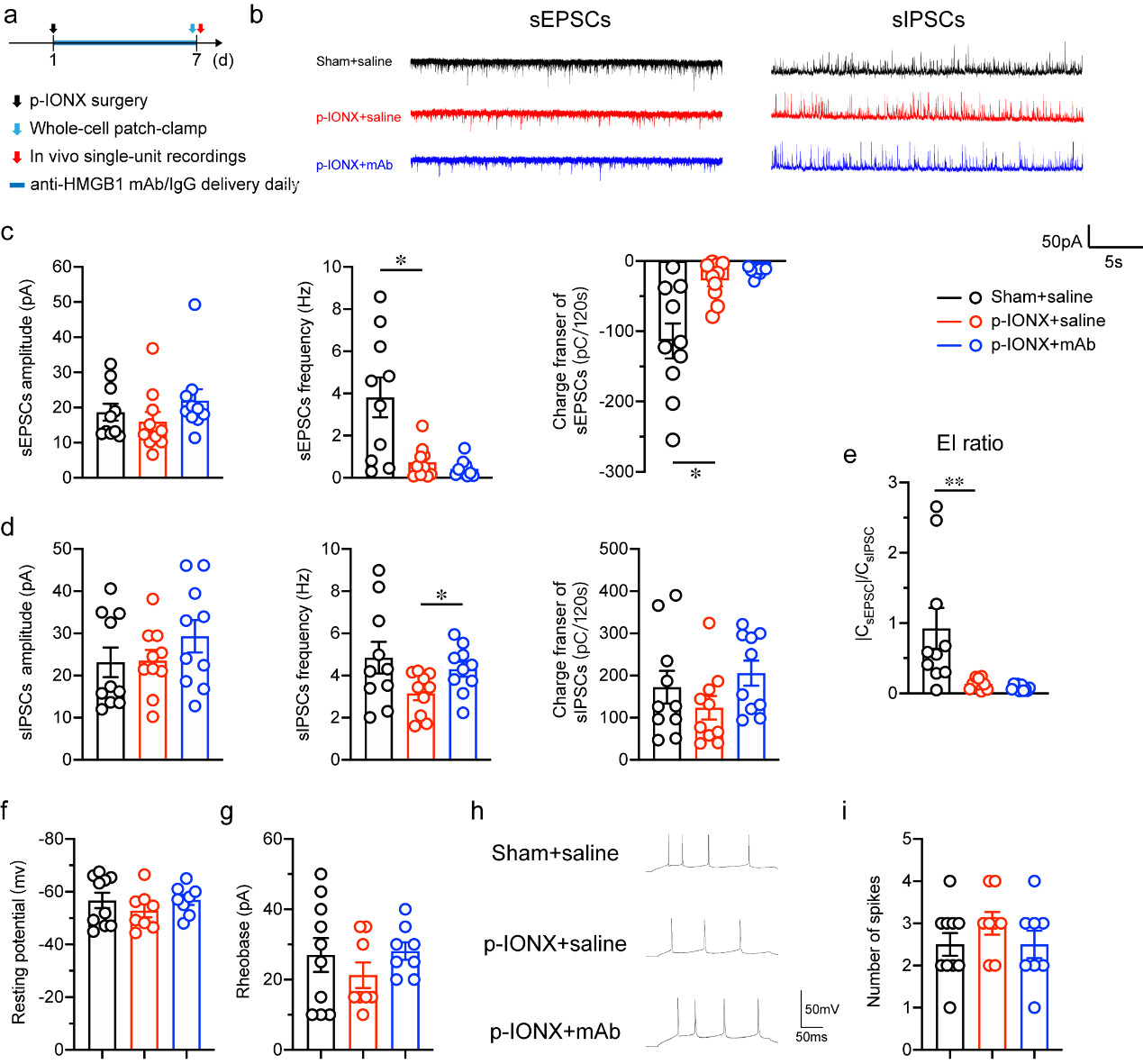


**Supplementary Fig. 5 Systemic anti-HMGB1 mAb did not affect the reduced activity of pyramidal neurons in mPFC layer 5/6 after p-IONX in MRL/MPJ mice. a** Schedule of experimental procedures. **b** Example of sEPSCs and sIPSCs in sham + saline, p-IONX+ saline and p-IONX+ anti-HMGB1 mAb groups. **c-d** Amplitude, frequency and charge transfer of sEPSCs (**c**) and sIPSCs (**d**). **e** Quantification of E/I ratio. **f** Resting potential. **g** Rheobase. **h-i** Representative traces of action potential firing (**h**) and number of spikes (**i**) evoked by injecting current at two-fold of rheobase. * and ** indicate *P <* 0.05 and 0.01, respectively, compared with the indicated groups, unpaired *t* test or Mann-Whitney test. *n* = 9-10 neurons from 4 mice/group.


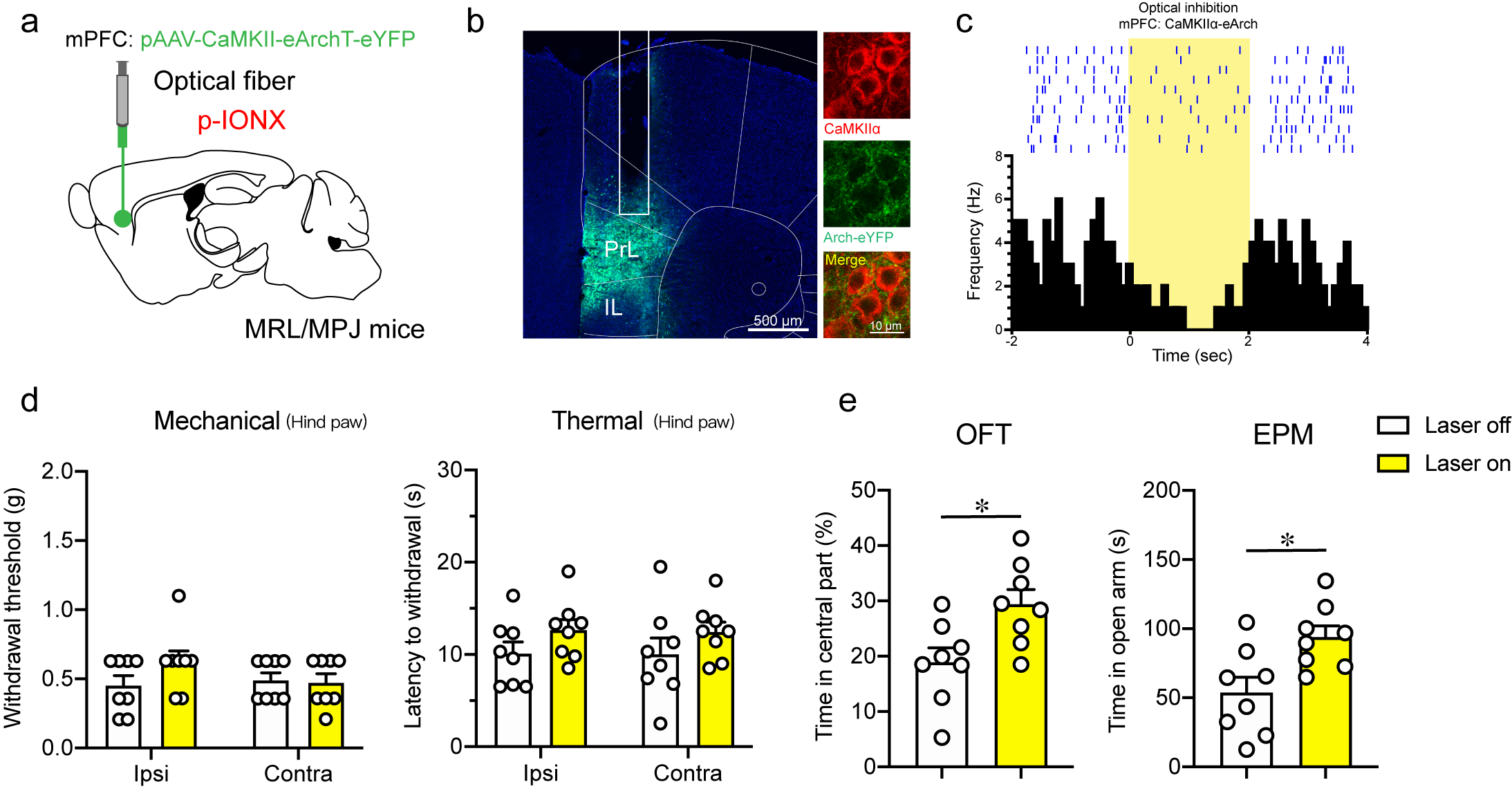


**Supplementary Fig. 6 Optogenetic inhibition of mPFC pyramidal neurons alleviated anxiety after p-IONX. a** Schematic drawing of sites for virus injection and optical fiber placement. **b** Example photomicrographs showing the Arch-eYFP in mPFC (left) and colocalization of eYFP and CaMKIIα, a marker of glutamatergic neurons (right). **c** Example neuron in the mPFC that deceased its firing frequency in response to yellow laser stimulation. **d** Paw withdrawal thresholds to mechanical stimulation and noxious heat stimulation. **e** Anxiety-like behaviors measured by OFT and EPM tests on D14 after surgery. ** indicates *P <* 0.01, compared with the laser off group, unpaired *t* test. *n* = 8-10/group.
